# Rational Design of Dual-Domain Binding Inhibitors for *N*-Acetylgalactosamine Transferase 2 with Improved Selectivity over the T1 and T3 Isoforms

**DOI:** 10.1021/jacsau.4c00633

**Published:** 2024-09-11

**Authors:** Ismael Compañón, Collin J. Ballard, Erandi Lira-Navarrete, Tanausú Santos, Serena Monaco, Juan C. Muñoz-García, Ignacio Delso, Jesus Angulo, Thomas A. Gerken, Katrine T. Schjoldager, Henrik Clausen, Tomás Tejero, Pedro Merino, Francisco Corzana, Ramon Hurtado-Guerrero, Mattia Ghirardello

**Affiliations:** † Department of Chemistry and Instituto de Investigación en Química de la Universidad de La Rioja, Universidad de La Rioja, Logroño 26006, Spain; ‡ Department of Biochemistry, Case Western Reserve University, 2109 Adelbert Rd, Cleveland, Ohio 44106, United States; § Department of Cellular and Molecular Medicine, Faculty of Health Sciences, Copenhagen Center for Glycomics, University of Copenhagen, Copenhagen 2200, Denmark; ∥ School of Pharmacy, University of East Anglia, Norwich Research Park, NR4 7TJ Norwich, U.K.; ⊥ Instituto de Investigaciones Químicas, Consejo Superior de Investigaciones Científicas and Universidad de Sevilla, Avenida Américo Vespucio, 49, Sevilla 41092, Spain; # Departments of Biochemistry and Chemistry, Case Western Reserve University, 2109 Adelbert Rd, Cleveland, Ohio 44106, United States; ¶ Department of Organic Chemistry, Faculty of Sciences, University of Zaragoza, Zaragoza 50009, Spain; ∇ Institute of Chemical Synthesis and Homogeneous Catalysis, University of Zaragoza-CSIC, Zaragoza 50009, Spain; ○ Institute for Biocomputation and Physics of Complex Systems, University of Zaragoza, Zaragoza 50018, Spain; ⧫ Fundación ARAID, Zaragoza 50018, Spain

**Keywords:** *N*-acetylgalactosamine transferase, glycosyltransferase, inhibitor, glycopeptide, GalNAc, molecular dynamics, STD NMR

## Abstract

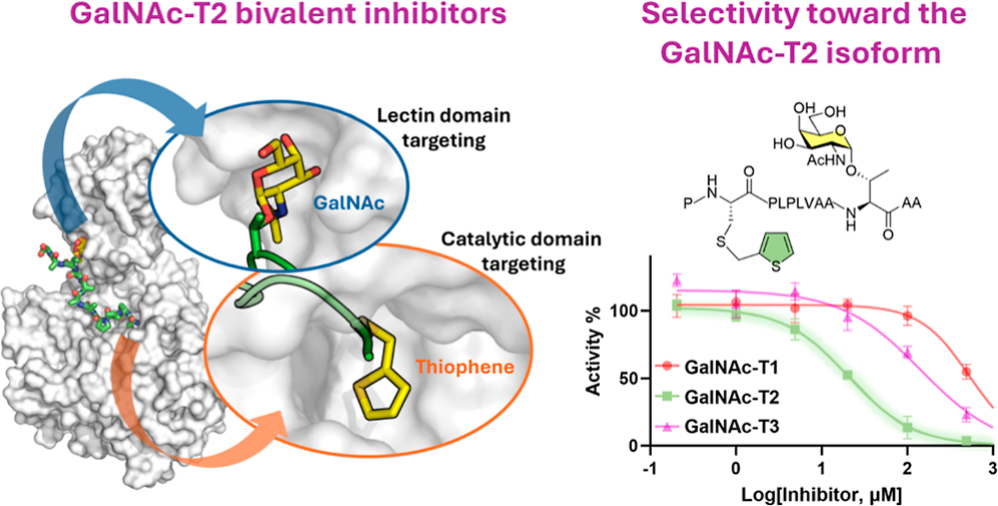

The GalNAc-transferase (GalNAc-T) family, consisting of 20 isoenzymes, regulates the *O*-glycosylation process of mucin glycopeptides by transferring GalNAc units to serine/threonine residues. Dysregulation of specific GalNAc-Ts is associated with various diseases, making these enzymes attractive targets for drug development. The development of inhibitors is key to understanding the implications of GalNAc-Ts in human diseases. However, developing selective inhibitors for individual GalNAc-Ts represents a major challenge due to shared structural similarities among the isoenzymes and some degree of redundancy among the natural substrates. Herein, we report the development of a GalNAc-T2 inhibitor with higher potency compared to those of the T1 and T3 isoforms. The most promising candidate features bivalent GalNAc and thiophene moieties on a peptide chain, enabling binding to both the lectin and catalytic domains of the enzyme. The binding mode was confirmed by competitive saturation transfer difference NMR experiments and validated through molecular dynamics simulations. The inhibitor demonstrated an IC_50_ of 21.4 μM for GalNAc-T2, with 8- and 32-fold higher selectivity over the T3 and T1 isoforms, respectively, representing a significant step forward in the synthesis of specific GalNAc-T inhibitors tailored to the unique structural features of the targeted isoform.

## Introduction

Glycosylation is one of the most complex and fundamental types of post-translational modification (PTM) occurring in cells,^1^ and it plays a critical role in structural, metabolic, and regulatory cell functions.^2^ This process is orchestrated by glycosyltransferase enzymes, and its dysregulation has emerged as a hallmark of numerous diseases such as cancer, where the aberrant glycosylation is implicated in tumor progression, immune evasion, and metastasis.^3−7^ Mucin-type *O*-glycosylation stands out as one of the most abundant and diverse types of protein *O*-glycosylation. It normally targets serine (Ser) and threonine (Thr) residues and is characterized by the display of complex glycans on tandem repeats of mucin proteins.^8^ The initial step in mucin *O*-glycosylation involves the transfer of an *N*-acetylgalactosamine (GalNAc) unit from uridine diphosphate-GalNAc (UDP-GalNAc) donors to Ser/Thr residues of acceptor substrates. In humans, this process is achieved by a family of up to 20 GalNAc-transferase (GalNAc-Ts) isoenzymes in an organized manner.^9^ These GalNAc-Ts are localized in the Golgi apparatus and are distributed with varying expression levels across all organs and tissues.^10^

In the past 15 years, numerous studies have revealed specific correlations between the dysregulation of various GalNAc-Ts and the progression of human diseases. For instance, GalNAc-T3 has been implicated in tumoral calcinosis,^11^ where it plays a coregulatory role in phosphate homeostasis.^12^ GalNAc-T11 has been associated with chronic kidney decline due to its role in the glycosylation of low-density lipoprotein receptors and related receptors, including LRP2.^13−15^ GalNAc-T6 overexpression showed to promote pancreatic^16^ and colorectal cancer^17^ development, while GalNAc-T7 upregulation correlated with tumor growth progression in prostate cancer patients.^18^

Recently, through GWAS and animal knockout models, GalNAc-T2 dysfunction has been implicated in cholesterol and triglyceride metabolism.^19,20^ Additionally, through its role in the GalNAcylation process of the insulin receptor, GalNAc-T2 has been identified as a key modulator of insulin signaling.^21^ Furthermore, lack of GalNAc-T2 expression has been established as a congenital disorder of glycosylation (GALNT2-CDG) linked to neurodevelopmental delays, and to the insurgence of brain abnormalities.^20^

These findings underscore the high value of GalNAc-Ts as promising drug targets under severe human conditions. However, the development of novel drugs targeting specific GalNAc-Ts represents a formidable challenge due to the difficulty in achieving potent and selective inhibitor against the targeted isoform.^22−24^ This is in contrast to protein phosphorylation, the most frequent type of PTM, which has seen significant success in drug development, with more than 70 approved drugs since 2001.^25^ All GalNAc-T isoenzymes utilize UDP-GalNAc as an activated sugar-nucleotide donor, and most of the family members showed some degree of redundancy in their protein and glycoprotein acceptor substrates.^10,26^ Therefore, inhibitors based on the sole structure of the donor or acceptor substrates are likely to exhibit promiscuous activity on a broad set of GalNAc-Ts. Several attempts into proving GalNAc-Ts druggability have been made, from the screening of libraries^24,27−29^ to the specific design of uridine derivatives.^24,27,30,31^ However, the high degree of similarity between GalNAc-T isoforms limited the development of specific inhibitors. The exception being T3Inh-1 that can act against GalNAc-T3 and showed no activity against the T2 and T6 variants, where it was used to lower the FGF23 levels in mice for chronic kidney disease treatment.^28^ Bioengineering approaches also demonstrated to be successful for selective GalNAc-Ts inhibition. For instance, engineered GalNAc-Ts through the “bump-and-hole” strategy using sterically hindered UDP-GalNAc variants has been reported.^32,33^ However, this strategy cannot be employed with nonengineered GalNAc-Ts in natural settings.

**Figure 1:**
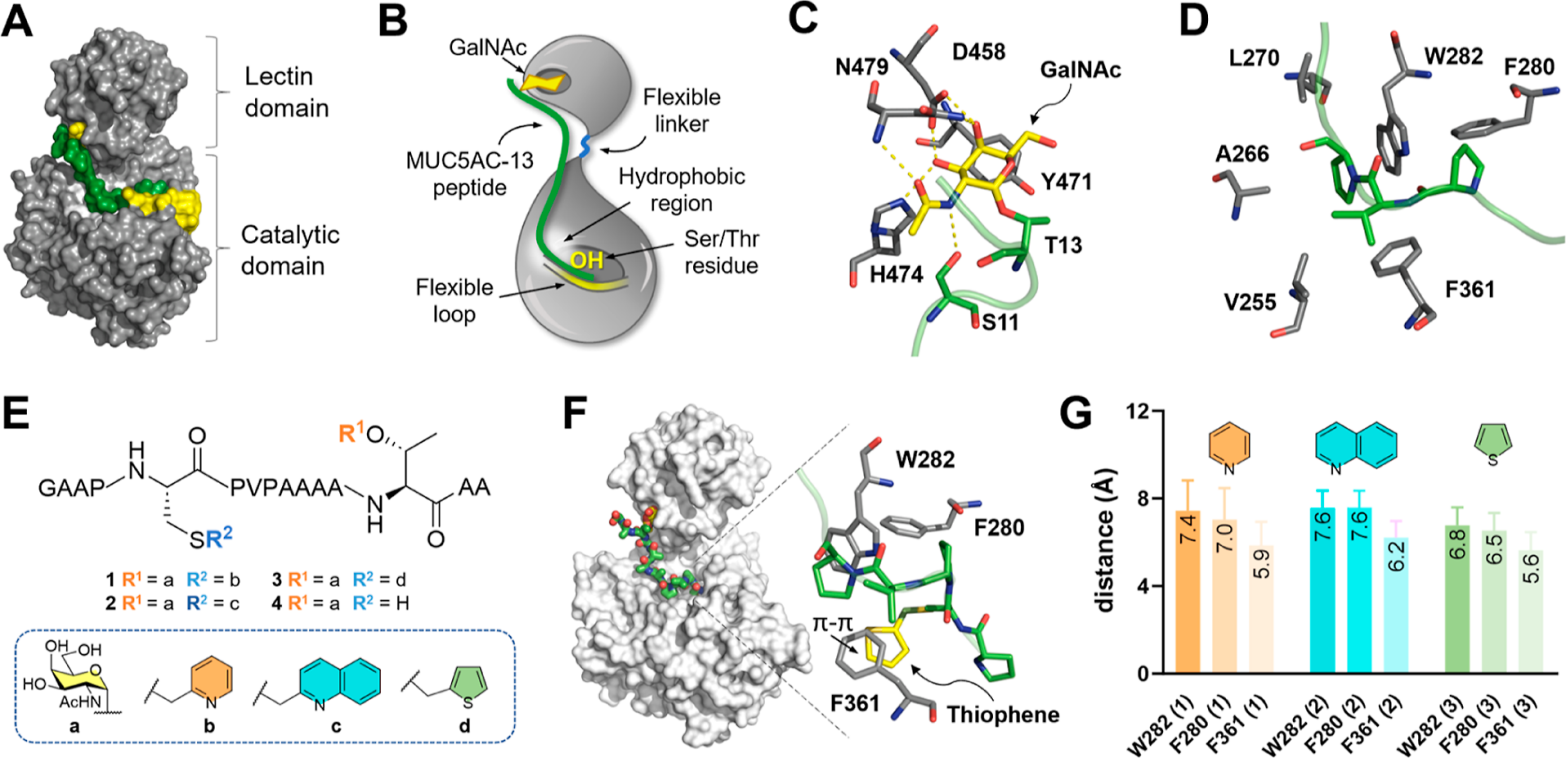
(A) Structure of GalNAc-T2 in complex with glycopeptide MUC5AC-13 and UDP. (B) Schematic representation of the GalNAc-T2 regions and of the binding mode with MUC5AC-13 glycopeptide. (C) Binding mode of the MUC5AC-13 GalNAc moiety (yellow) with GalNAc-T2 lectin pocket (gray). (D) Binding mode of MUC5AC-13 PVP residues (green) with the hydrophobic region of GalNAc-T2 (gray). PDB ID: 5AJP. (E) Chemical structure of inhibitors **1**–**4**. (F) Representative frame derived from 1.0 μs molecular dynamics (MD) simulation of compound **3** with GalNAc-T2 and expansion on the interactions between the thiophene moiety and residues W282, F280, and F361. (G) Average distance between the aromatic moieties of **1**, **2**, and **3** with the residues W282, F280, and F361 of GalNAc-Ts calculated through 1.0 μs MD simulations; data represent mean + SD (standard deviation).

In an attempt to achieve a selective inhibition toward GalNAc-T2, we have prepared a series of glycopeptides rationally designed on the basis of structural knowledge gathered on this GalNAc-T isoform.^34−37^ All GalNAc-Ts enzymes possess a lectin domain responsible for recognition and binding to a previously glycosylated position of the glycopeptide acceptor substrate (Figure 1A,B). This domain is connected through a flexible linker to the catalytic domain, which binds the UDP-GalNAc donor substrate and transfers the activated sugar unit to hydroxyl residues of the acceptor substrate. A flexible loop present nearby the catalytic pocket promotes the binding of the donor substrate, and its dynamics regulate the switch between active and inactive conformation of the enzyme.^34^ GalNAc-Ts can glycosylate in a lectin-independent or lectin-dependent manner. The latter can be classified into two main subfamilies depending on the preference to glycosylate a position within a short-range (1 to 3 residues) or long-range (6 to 17 residues) to a previously glycosylated position.^10,38^ The dynamics of the interdomain flexible linker and the enzyme electrostatic surface potential, dictate the preference to glycosylate long- or short-range positions toward the N- or C-terminus direction of the acceptor chain.^36,39^ These structural features are likely used by the GalNAc-T family to promote mucin glycosylation in a hierarchical and organized manner. Previous studies reported the preference of GalNAc-T3/T4/T6/T12 to glycosylate long-range C-terminal positions from a prior N-terminal GalNAcylated residue. On the contrary, the GalNAc-T2/T14 isoforms preferably glycosylate toward the N-terminal direction,^26^ and GalNAc-T1 is capable of glycosylating with both preferences.^40^

In order to achieve selectivity over the target T2 isoform, we devised the synthesis of chemically modified bivalent glycopeptides capable of targeting both the lectin and catalytic domain of the enzyme at the same time. This family of inhibitors features a GalNAc moiety at the C-terminus, which enables binding to the lectin domain as for natural substrates (Figure 1C), and an aromatic moiety placed 7 residues away toward the N-terminus position. We hypothesized that the aromatic moiety would interact with the hydrophobic region placed at the hinge between the catalytic domain and the flexible loop (Figure 1D), disrupting the dynamics of the loop during the substrate recognition process and inhibiting GalNAc-T2.

## Results and Discussion

MD techniques were used to screen different aromatic moiety candidates to improve the binding of a model glycopeptide to the hydrophobic region of GalNAc-T2.

The model peptide sequence GAAPĈPVPAAAAT*AA was used to screen different aromatic substituents, where Ĉ and T* represent an *S*-alkylated cysteine (Cys) and a *O*-GalNAcylated Thr, respectively. A simple alanine sequence was used as a spacer between T*, whereas the PV motif before was maintained as in the natural MUC1 acceptor substrate sequence to promote hydrophobic interaction with the aromatic region in proximity to the flexible loop hinge.

Three glycopeptide models bearing a pyridine (**1**), a quinoline (**2**), and a thiophene (**3**) aromatic substituent at Ĉ (Figure 1E) were studied through 1.0 μs MD simulations. The AMBER 22 software package was used and implemented with the appropriate force fields (see Supporting Information, Section 1).^41^ All complexes were generated using the crystal structure of the active form of GalNAc-T2 in complex with UDP and a MUC5AC-like glycopeptide as the initial structure (PDB ID: 5AJP).^35^

According to these calculations, compound **3** (Figure 1F,G), with a thiophene moiety, can interact more tightly with the enzyme than compounds **1** and **2**, as inferred from the reduced distances calculated from the simulations for this aromatic moiety and the side chains of F280, W282, F280, and F361 of GalNAc-T2 (see Supporting Information, Figure S1 for representative frames of the MD simulation with compounds **1–3**). To validate our hypothesis, glycopeptides **2** and **3** were synthesized and tested against GalNAc-T2.

**Figure 2:**
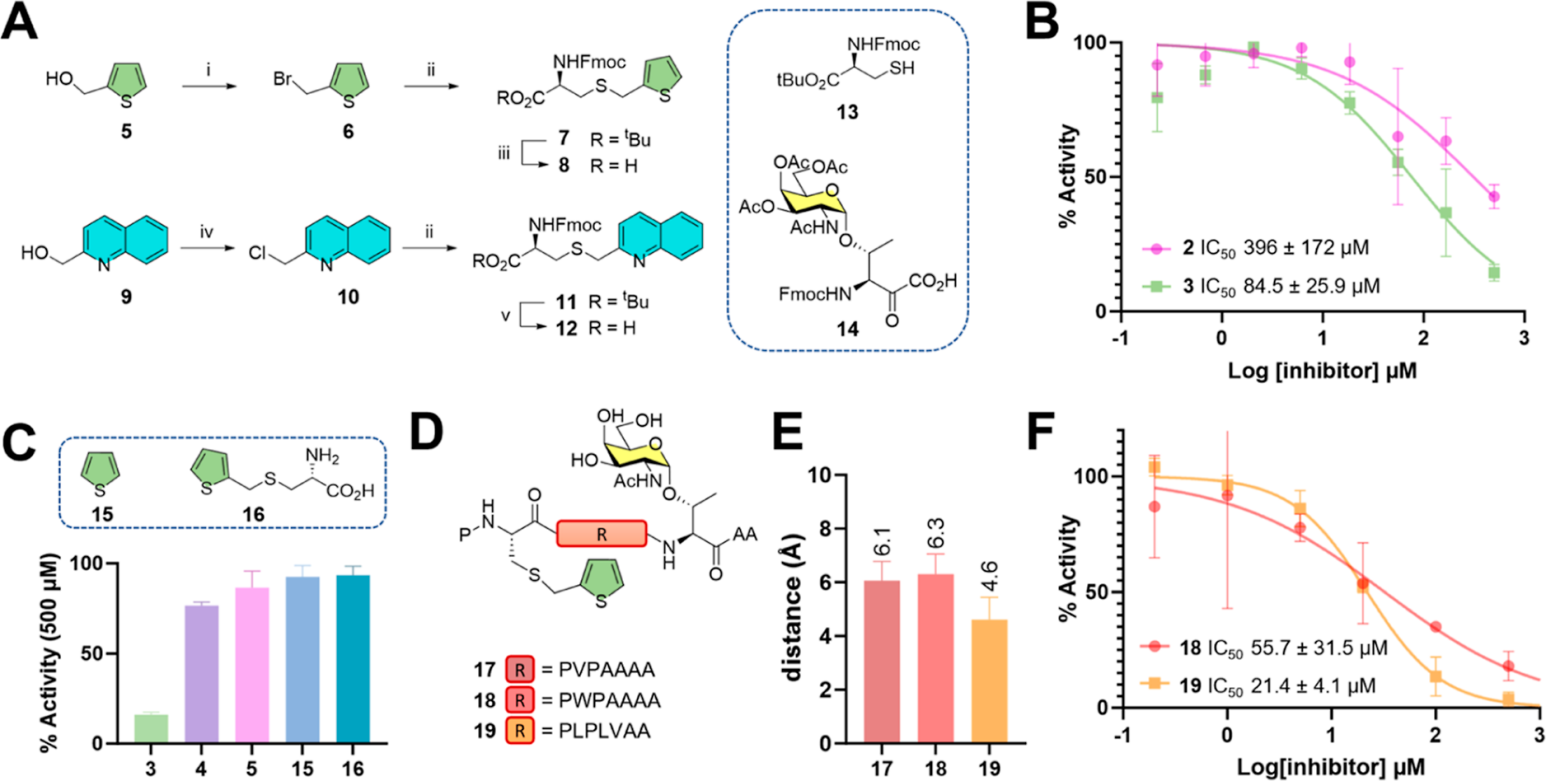
(A) Synthetic approach for the synthesis of building blocks **8** and **12**. Reagent and conditions: (i) HBr (33% in AcOH), Et_2_O, 0 °C to rt, 16 h, 96%; (ii) **13**, NaHCO_3_, Bu_4_NBr, H_2_O, EtOAc, 24 h, rt, 82%; (iii) EDT, TFA, DCM, 0 °C to rt, 1 h, 81%; (iv) SOCl_2_, DCM, 0 °C to rt, 1 h, 85%; and (v) TFA, DCM, 0 °C to rt, 2 h, 87%. (B) Relative inhibitory activity % of compounds **2** and **3** against GalNAc-T2 using MUC1a as acceptor substrate. (C) Relative inhibitory activity % of compounds **3**, **4**, **5**, **15**, and **16** at a normalized concentration of 500 μM against GalNAc-T2 using MUC1a as acceptor substrate; data represent mean + SD. (D) Chemical structure of inhibitors **17–19**. (E) Average distance between the thiophene moiety of **17**, **18**, and **19** with residue F361 of GalNAc-Ts calculated through 1.0 μs MD simulations; data represent mean + SD (standard deviation). (F) Relative inhibitory activity % of compounds **18** and **19** against GalNAc-T2 using MUC1a as acceptor substrate.

The thiophene derivative was prepared starting from commercially available 2-thiophenemethanol **5**, which was activated with HBr to provide bromide **6** (Figure 2A). Compound **6** was then reacted under mild basic conditions with the Cys derivative **13**(42) to give protected thiophene-Cys building block **7**. Finally, cleavage of the *t*-Bu protecting group with TFA furnished **8** in a good overall yield (Figure 2A). The quinoline derivative was prepared following a similar strategy, starting from commercially available 2-quinolinylmethanol **9**, which was converted into chloride **10** using SOCl_2_, and then reacted with **13** providing derivative **11**. Finally, *t*-Bu cleavage with TFA afforded quinoline-Cys building block **12** in good overall yields (Figure 2A). The Fmoc-protected amino acid α-*O*-GalNAc-Thr **14** was prepared as described in previously reported procedures.^43^ Glycopeptides **2** and **3** were then prepared through Fmoc-based solid phase peptide synthesis, and the target inhibitors were tested against GalNAc-T2.

The inhibition activity of glycopeptides **2** and **3** was determined using a radiometric assay using a labeled UDP-[^3^H or ^14^C]-GalNAc against a model peptide substrate MUC1a with sequence AHGVTSAPDTR, which was demonstrated to be a general substrate accepted by a wide range of GalNAc-Ts, including the T2 isoform.^38^ The glycosylation products were isolated from the radiolabeled donors through chromatographic techniques and analyzed by scintillation counting. The analysis demonstrated that both glycopeptides **2** and **3** were able to inhibit GalNAc-T2, showing IC_50_ values of 396 ± 172 and 84.5 ± 25.9 μM for **2** and **3**, respectively (Figure 2B), in good agreement with the MD prediction.

To prove that the inhibition activity was the result of a bivalent interaction between the inhibitor and both the lectin and catalytic domain of GalNAc-T2, and was not the result of nonspecific interactions, we synthesized and tested peptide **4**, which retains the GalNAc and animo acid sequence of **3** but lacks the thiophene moiety (Figure 1E). Moreover, we measured the activity of a set of thiophene fragments, including compound **5**, thiophene **15**, and unprotected thiophene-Cys **16**, at a normalized concentration of 500 μM (Figure 2C). The assay demonstrated that only glycopeptide **3** could induce a relevant inhibition of the GalNAc-T2 activity while the whole set of thiophene fragments and peptide **4** were unable to inhibit the enzyme. This demonstrated that the aromatic moiety is necessary to exert the inhibition activity, likely due to its binding to the catalytic domain of the enzyme. However, the thiophene fragments alone did not act as inhibitors, suggesting that the two moieties of the bivalent glycopeptides were required for an optimal inhibition.

Next, we investigated the possibility to achieving a stronger inhibition activity through the modification of the peptide sequence connecting the PĈP motif to the T* residue.

Using MD simulations, we screened a set of bivalent glycopeptides where the amino acid sequence was rationally modified to improve the π–π interaction between the thiophene moiety of **3** and the GalNAc-T2 F361 residue. We modeled three different variants **17**, **18**, and **19** (Figure 2D) using amino acidic residues presenting nonpolar side chains next to the PĈP motif. This feature was chosen with the objective to improving the interactions between the nonpolar side chains and the hydrophobic region nearby the flexible loop of the enzyme.

The analysis of the distance between the thiophene moiety of glycopeptides **17–19** and the F361 residue of GalNAc-T2, derived from MD simulations, rendered compounds **18** and **19** as the weakest and the strongest ligands, respectively (Figure 2E). These findings aligned with the experimentally measured IC_50_ values. Compound **18** had an IC_50_ value of 55.7 ± 31.5 μM, while compound **19** had an IC_50_ value of 21.4 ± 4.1 μM (Figure 2F). These results demonstrate the potential of using MD simulations in the inhibitor design process.

To date, compound **19** represents one of the best inhibitors ever reported against GalNAc-T2, with a comparable potency to luteolin (IC_50_ 14.7 μM), which was discovered through the high-throughput screening of libraries of compounds^29^ and a mimetic of the UDP-GalNAc donor substrate referred to as compound 1–68A (IC_50_ 15.0 μM).^27^ However, these compounds were not tested against different GalNAc-Ts and are likely to have broad activity against various isoforms. This is expected, especially in the case of compounds 1–68A, which targets the UDP-GalNAc binding site that is a common glycoside donor shared by the whole GalNAc-T family.

**Figure 3:**
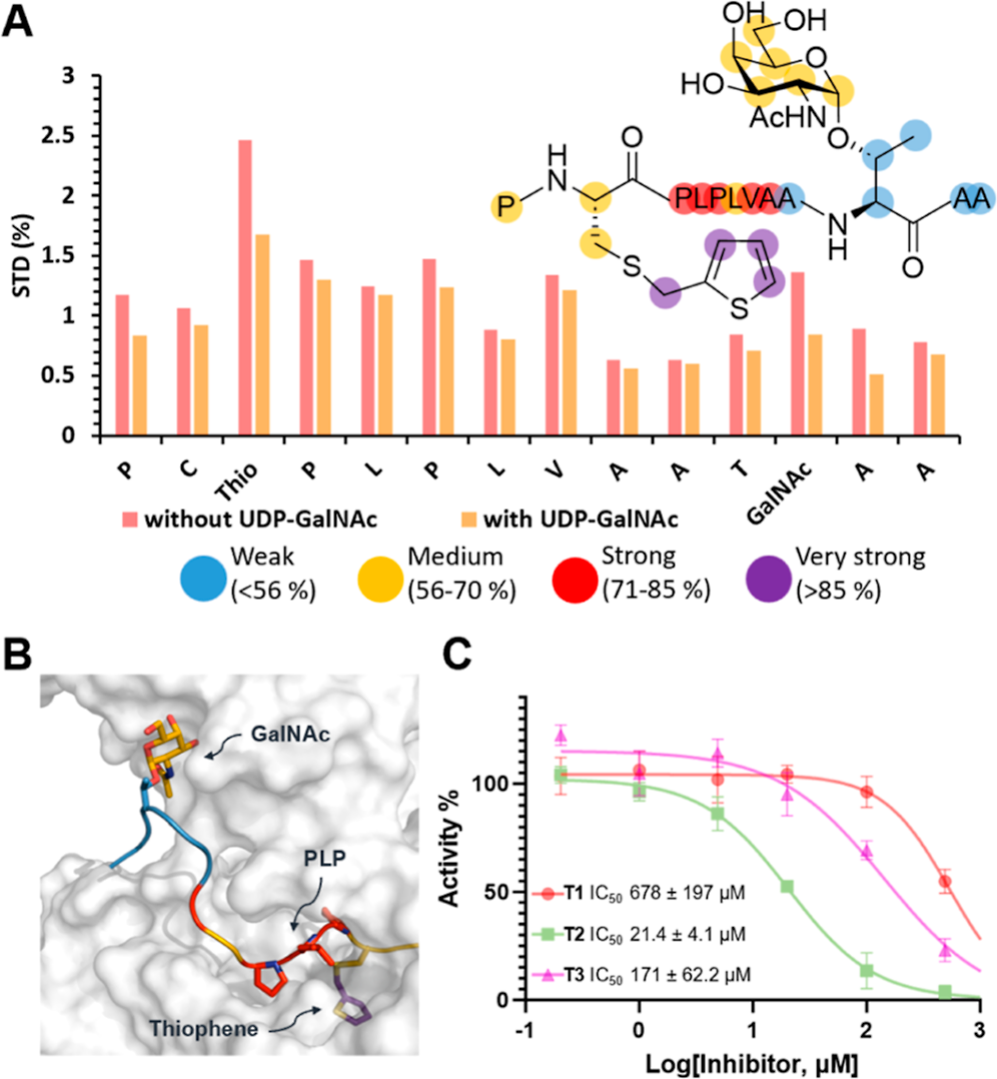
(A) STD NMR binding epitope map of **19** upon binding to GalNAc-T2, analyzed as an average per residue, due to significant proton signal overlapping. Colors represent different degrees of contact, from light blue to purple, as shown in the legend, and STD NMR competition experiments performed adding UDP-GalNAc to a sample containing **19** and GalNAc-T2. (B) Representative MD frame of **19** in complex with GalNAc-T2. Color code of **19** corresponds to the degree of contact with GalNAc-T2 as for Figure 3A. (C) Relative inhibitory activity % of compound **19** against GalNAc-T1, -T2, and -T3 using MUC1a as acceptor substrate.

To shed light on the binding mode of **19** with GalNAc-T2, we conducted a series of saturation transfer difference (STD) NMR experiments. This technique allows us to map the parts of the inhibitor in close contact with the enzyme, since the highest STD intensities correlate with the closest ligand–protein contacts in the bound state. As a result, STD NMR provides so-called ligand binding epitope mapping depicting the most important moieties responsible for binding. Additionally, to gather information on the location of binding, we used the STD NMR competition of **19** with UDP-GalNAc, which is known to bind in the catalytic domain of the protein (Figure 3A). Due to significant signal overlap, we could provide a reliable analysis only based on averages per residue along the peptide, though the information gained is still relevant. Remarkably, the binding epitope map showed that the thiophene-bearing N-terminal end of the peptide received the largest amount of saturation from the enzyme. In fact, the thiophene residue is the moiety showing the closest contacts with the GalNAc-T2 surface in the bound state, suggesting that this region of thiophene glycopeptide **19** is fundamental to establishing favorable contacts with the enzyme. On the other hand, the GalNAc moiety also showed significant contacts with the surface of GalNAc-T2 in the bound state, despite the low STD intensities shown by the adjacent alanine residues. This result is compatible with a bidentate binding where the GalNAc sugar ring fits in the binding site of the lectin domain, whereas the thiophene and adjacent residues are located in the catalytic domain’s binding site. The STD NMR data is correlated with the MD simulation, where the thiophene and the PLP motif are deeply inserted into the groove of the GalNAc-T2 catalytic site, establishing close contacts with the enzyme (Figure 3B). Similarly, the GalNAc moiety showed close contacts with the enzyme’s lectin domain, while the C-terminal AA motif was exposed to the solvent in good agreement with the STD NMR results. The competition experiment with UDP-GalNAc showed a general decrease of the STD intensities with major changes occurring for the thiophene moiety, demonstrating that the inhibitors compete with the donor substrate too by interacting through the thiophene moiety with the catalytic site. These results corroborated that all of the key components of compound **19**, i.e., the GalNAc moiety, the nonpolar amino acid sequence, and the thiophene moiety, are involved in the binding to GalNAc-T2.

Finally, we determined the potential selectivity of inhibitor **19**, which was designed based on structural features of GalNAc-T2, known to prefer glycosylating N-terminal residues from a prior C-terminal GalNAcylated position.^26^ This potential selectivity was assessed over two enzymatic models using GalNAc-T3,^26^ which exhibits the opposite preference, and GalNAc-T1, which can glycosylate with both preferences.^41^ Moreover, these three enzymes hold high biological relevance, representing the most abundant GalNAc-Ts, which are widely expressed in most tissues.^44^ We measured and compared the inhibitory activity of **19** against GalNAc-T1, T2, and T3 enzymes and demonstrated the high degree of selectivity toward the T2 isoform with IC_50_ values of 678 ± 197 μM, 21.4 ± 4.1 μM, and 170.8 ± 62.2 μM against GalNAc-T1, -T2, and -T3, respectively (Figure 3C). This corresponds to an 8x more potent inhibitory activity against GalNAc-T2 compared to T3, and a 32x higher selectivity with respect to the T1 isoform. Moreover, it was possible to calculate the kinetic parameters of the enzymatic inhibition for the three GalNAc-Ts (see Supporting Information in Table S1 in Section 4.4). The comparison of the K_I_ values calculated for both the acceptor and donor substrates showed 31× and 7× higher potencies for GalNAc-T2 inhibition with respect to GalNAc-T1 and T3, respectively. These findings demonstrate a strong correlation between the results, confirming the selectivity of glycopeptide **19** for inhibiting GalNAc-T2.

In summary, we identified and synthesized compound **19** as the first selective GalNAc-T2 inhibitor reported so far. It is worth noting that the GalNAc-T3/T4/T6/T12 isoforms prefer the glycosylate long-range C-terminal position; therefore, a certain degree of selectivity of **19** toward the T2 over T3 isoform was expected. Remarkably, **19** was, with unprecedent selectivity, able to inhibit the T2 over the T1 isoform, which are both capable to operate toward long-range N-terminal positions.^26^ This demonstrates the great potential of our strategy toward the synthesis of specific GalNAc-T inhibitors.

## Conclusions

In this study, a structure-guided rational design approach was used to synthesize a GalNAc-T2 inhibitor with improved potency over other isoforms by targeting both the lectin and catalytic domains of the enzyme. Compound **19** demonstrated how the dual targeting of the lectin and catalytic domain of GalNAc-T2 leads to an effective inhibition of the enzyme with an IC50 of 21 μM. Compared to the T1 and T3 isoforms compound **19** showed a 32- and 8-times greater selectivity toward the targeted T2 isoform, demonstrating how the MD-assisted rational design of bivalent inhibitors improves the selective inhibition of a single isoform. Further studies in cellular models to assess the effectiveness of compound **19** in the inhibition of GalNAc-T2 are required before its implementation in biological settings. However, this versatile strategy could be effectively exploited for the synthesis of selective inhibitors of other GalNAc-Ts, setting the stage for future drug development oriented toward glycan-based therapeutics.

## Supporting Information Available

MD simulation, chemical synthesis, STD NMR studies, and biochemical studies (PDF)
