# Supplementary material for: Rational Design of Dual-Domain Binding Inhibitors for N-Acetylgalactosamine Transferase 2 with Improved Selectivity over the T1 and T3 Isoforms
Source: JACS Au. 2024 Sep 11;4(9):3649–56. doi: 10.1021/jacsau.4c00633 (PMC11423303; doi:10.1021/jacsau.4c00633)
Supplement: Supplementary file 1 — au4c00633_si_001.pdf [file au4c00633_si_001.pdf]

# SUPPORTING INFORMATION

# Rational Design of Dual-Domain Binding Inhibitors for *N*-acetylgalactosamine Transferase 2 with Improved Selectivity Over the T1 and T3 isoforms

Ismael Compañón,[a] Collin J. Ballard,[b] Erandi Lira-Navarrete,[c] Tanausú Santos,[a] Serena Monaco,[d] Juan Carlos Muñoz-García,[d, e] Ignacio Delso,[d] Jesus Angulo,[d, e] Thomas A. Gerken,[b, f] Katrine T. Schjoldager,[c] Henrik Clausen,[c] Tomás Tejero,[g, h] Pedro Merino,[g, i] Francisco Corzana,[a] Ramon Hurtado-Guerrero[c, i, j] and Mattia Ghirardello*[a]

[a] Department of Chemistry and Instituto de Investigación en Química de la Universidad de La Rioja (IQUR), Universidad de La Rioja, Logroño, 26006, Spain.
[b] Department of Biochemistry, Case Western Reserve University, 2109 Adelbert Rd, Cleveland, OH, 44106, USA.
[c] Department of Cellular and Molecular Medicine, Faculty of Health Sciences, Copenhagen Center for Glycomics, University of Copenhagen, Copenhagen, Denmark.
[d] School of Pharmacy, University of East Anglia, Norwich Research Park, NR4 7TJ Norwich, UK.
[e] Instituto de Investigaciones Químicas (IIQ), Consejo Superior de Investigaciones Científicas and Universidad de Sevilla, Avenida Américo Vespucio, 49, Sevilla, 41092, Spain.
[f] Departments of Biochemistry and Chemistry, Case Western Reserve University, 2109 Adelbert Rd, Cleveland, OH, 44106, USA.
[g] Department of Organic Chemistry. Faculty of Sciences, University of Zaragoza, Zaragoza, Spain.
[h] Institute of Chemical Synthesis and Homogeneous Catalysis (ISQCH), University of Zaragoza-CSIC, Zaragoza, Spain.
[i] Institute for Biocomputation and Physics of Complex Systems (BIFI), University of Zaragoza, Zaragoza, Spain.
[j] Fundación ARAID, Zaragoza, Spain.

* correspondence to be addressed to: mattia.ghirardello@unirioja.es.

## Table of Contents

**1. MOLECULAR DYNAMICS SIMULATIONS ........ S1**

**2. CHEMICAL SYNTHESIS ........ S3**
2.1 Material and methods ........ S3
2.2. Synthesis of thiophene building block (8) ........ S3
2.3. Synthesis of quinoline building block (12) ........ S5
2.4 Solid phase peptide synthesis (2,3,4,18,19) ........ S7
2.5. NMR Spectra ........ S13

**3. STD NMR STUDIES ........ S17**

**4. BIOCHEMICAL STUDIES ........ S19**
4.1. Reagents and substrates ........ S19
4.2. GalNAc-Ts expression ........ S19
4.3. Inhibition reactions ........ S19

**5. SUPPLEMENTARY REFERENCES ........ S21**

## 1. MOLECULAR DYNAMICS SIMULATIONS

The simulations were carried out with AMBER 22 package[1] implemented with ff14SB,[2] GAFF,[3] and GLYCAM06[4] force fields. The parameters and charges for the unnatural amino acids were generated with the antechamber module of AMBER, using GAFF force field and AM1-BCC[5] method for charges. The crystal structure of the active form of GalNAc-T2 in complex with UDP and a MUC5AC-like glycopeptide[6] was used in these studies. Each molecule was then immersed in a water box with a 10 Å buffer of TIP3P water molecules.[7] The system was neutralized by adding explicit counter ions. A two-stage geometry optimization approach was performed. The first stage minimizes only the positions of solvent molecules, and the second stage is an unrestrained minimization of all the atoms in the simulation cell. The systems were then gently heated by incrementing the temperature from 0 to 300 K under the constant pressure of 1 atm and periodic boundary conditions. Harmonic restraints of 30 kcal·$mol^{-1}$ were applied to the solute, and the Andersen temperature-coupling scheme was used to control and equalize the temperature. The time step was kept at 1 fs during the heating stages, allowing potential inhomogeneities to self-adjust. Long-range electrostatic effects were modeled using the particle-mesh-Ewald method.[8] An 8 Å cut-off was applied to Lennard-Jones interactions. Each system was equilibrated for 2 ns with a 2-fs time step at a constant volume and temperature of 300 K. Production trajectories were then run for additional 1.0 μs under the same simulation conditions.

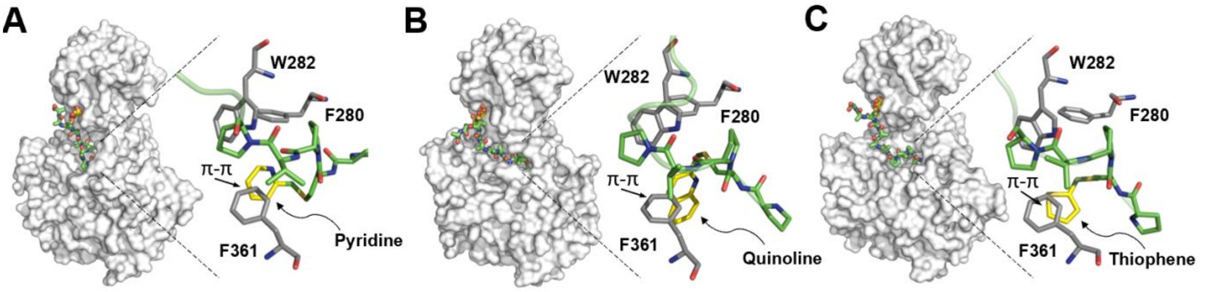


**Figure S1**. Representative frame derived from 1.0 μs MD simulation of compounds **1**-**3** with GalNAc-T2 and expansion on the interactions between the pyridine/quinoline/thiophene moieties (yellow) and residues W282, F280, and F361 (gray). **A**) Interactions between compound **1** and GalNAc-T2. **B**) Interactions between compound **2** and GalNAc-T2. **C**) Interactions between compound **3** and GalNAc-T2.

## 2. CHEMICAL SYNTHESIS

### 2.1 Material and methods

The reaction flasks and other glass equipment were heated in an oven at 130 °C overnight and assembled in a stream of Ar. All reactions were monitored by TLC on silica gel 60 F254; the position of the spots was detected with 254 nm UV light or by spraying with either 5 % ethanolic phosphomolybdic acid or potassium permanganate (1.5 g $KMnO_4$, 10 g $K_2CO_3$ and 1.25 mL of 10% NaOH in 100 mL of $H_2O$) solutions. Column chromatography was carried out in a Buchi 800 MPLC system or a Combiflash apparatus, using silica gel 60 microns and with solvents distilled prior to use. Purification by semipreparative HPLC (column Atlantis® DC18 5 µm, 19x100 mm, flow: 12.5 mL/min) was carried out in a Waters 515 pump with PDA detection. $^1H$ and $^{13}C$ NMR spectra were recorded on Bruker Avance 400 MHz or AVANCE II 300 MHz instruments in the stated solvent at 298 K. Chemical shifts are quoted in parts per million from residual solvent peak ($CDCl_3$: $^1H$: 7.26 ppm and $^{13}C$: 77.16 ppm) and coupling constants (J) given in Hertz. Multiplicities are abbreviated as: b (broad), s (singlet), d (doublet), t (triplet), q (quartet), m (multiplet) or combinations thereof. NMR assignments were made using standard 2D experiments. Optical rotations were taken on a JASCO DIP-370 polarimeter. Elemental analyses were performed on a Perkin Elmer 240B microanalyzer or with a Perkin-Elmer 2400 instrument. High resolution mass spectra were recorded on a QToF spectrometer equipped with an ESI source (microTOF-Q, Bruker Daltonik) using sodium formate as external reference.

### 2.2. Synthesis of thiophene building block (8)

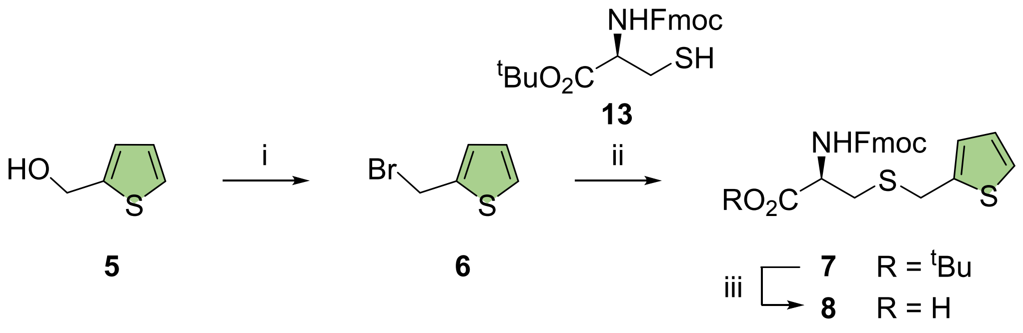


**Scheme S1**. Synthetic approach for the synthesis of building blocks **8**. Reagent and conditions: (i) HBr (33 % in AcOH), $Et_2O$, 0 °C to rt, 16 h, 96 %; (ii) **13**, $NaHCO_3$, $Bu_4NBr$, $H_2O$, EtOAc, 24 h, rt, 82 % (yield calculated on the limiting reagent **13**); (iii) TFA, DCM, 0 °C to rt, 2 h, 81 %.

## ***N*-Fmoc-*S*-methyl-2-thiophenyl-L-cysteine *tert*-Butyl Ester (7)**

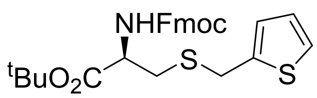

To a stirred solution of **5** (1.66 mL, 17.50 mmol) in anhydrous $Et_2O$ (50 mL) cooled to 0 °C under Ar atmosphere, HBr (33 % AcOH, 4.0 mL, 22.84 mmol) was added. The solution was stirred at room temperature for 16 hours, then diluted with ice cooled water (50 mL); and extracted with $Et_2O$ (2 x 25 mL). The combined organic phases were washed with saturated $NaHCO_3$ aqueous solution until neutral pH was reached and then with brine (50 mL). The organic phase was dried with anhydrous $MgSO_4$ and concentrated giving crude **6** (2.99 g, 96 %) as a transparent liquid that was directly used in the next step without further purification.

A solution of Tetrabutylammonium Bromide (2.18 g, 6.76 mmol) in saturated $NaHCO_3$ aqueous solution (10 mL) was added to a stirred solution of **13** (675 mg, 1.69 mmol) and **6** (600 mg, 3.37 mmol) in Ar bubbled EtOAc (10 mL); under Ar atmosphere and stirred vigorously for 24 h. After that time the mixture was diluted with EtOAc (100 mL) and washed with $H_2O$ (30 mL) and brine (30 mL). The organic phase was dried with anhydrous $MgSO_4$ and concentrated. The crude residue was purified by column chromatography on silica gel (Hexane/EtOAc, 1:0 to 4:1 v/v) to give **7** (684 mg, 82 %, calculated on the limiting reagent **13**) as a white solid.

**mp**: 75-77 °C. $[\alpha]_D^{20}$ = -27 (*c* 1.0, $CH_2Cl_2$). **$^1$H NMR** (400 MHz, $CDCl_3$) $\delta$ 7.81 – 7.74 (m, 2H, $H_{Fmoc}$), 7.67 – 7.59 (m, 2H, $H_{Fmoc}$), 7.44 – 7.38 (m, 2H, $H_{Fmoc}$), 7.32 (tt, *J* = 7.5, 1.5 Hz, 2H, $H_{Fmoc}$), 7.20 (dd, *J* = 5.1, 1.3 Hz, 1H, H-5$_{Thiophene}$), 6.94 (d, *J* = 3.6 Hz, 1H, H-3$_{Thiophene}$), 6.90 (dd, *J* = 5.1, 3.4 Hz, 1H, H-4$_{Thiophene}$), 5.60 (d, *J* = 7.9 Hz, 1H, NH), 4.52 (dt, *J* = 7.9, 5.2 Hz, 1H, C*H*$CH_2$), 4.41 (dq, *J* = 5.8, 3.4 Hz, 2H $CH_{2Fmoc}$), 4.25 (t, *J* = 7.2 Hz, 1H, $CH_{Fmoc}$), 3.96 (d, *J* = 4.2 Hz, 2H, C$H_2$-Thiophene), 3.04 – 2.85 (m, 2H, CHC$H_2$), 1.49 (s, 9H, $^t$Bu). **$^{13}$C APT NMR** (100 MHz, $CDCl_3$) $\delta$ 169.7 ($C_{COOt\text{-}Bu}$); 155.8 ($C_{Fmoc}$); 143.9 (C); 143.8 (C); 141.3 (C); 141.3 (C); 141.1 (C); 127.8 (2 $C_{Fmoc}$); 127.1 (2 $C_{Fmoc}$); 126.7 ($C_{Thiophene}$); 126.6 ($C_{Thiophene}$); 125.3 ($C_{Thiophene}$); 125.2 (2 $C_{Fmoc}$); 120.0 (2 $C_{Fmoc}$); 82.9 (C); 67.1 ($C_{Fmoc}$); 54.1 (*C*H$CH_2$); 47.2 ($C_{Fmoc}$); 34.0 (CH*C*$H_2$); 31.2 (*C*$H_2$-Thiophene); 28.0 (3 $C_{t\text{-}Bu}$). **Elemental analysis** calcd (%) for $C_{27}H_{29}NO_4S_2$: C, 65.43; H, 5.90; N, 2.83; O, 12.91; S, 12.94; found: C 66.28, H 5.73, N 2.81, S 12.57.

## ***N*-Fmoc-*S*-methyl-2-thiophenyl-L-cysteine (8)**

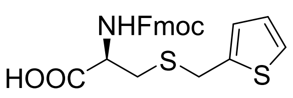

To a stirred solution of **7** (300 mg, 0.61 mmol) in anhydrous DCM (4 mL) cooled to 0 °C EDT (760 µL, 9.07 mmol) and TFA (4.0 mL, 52.27 mmol) were added. The resulting solution was allowed to rise to room temperature, stirred for 1 hour, and then concentrated under reduced pressure. The residue was previously purified by column chromatography on silica gel (DCM/MeOH, 1:0 → 99:1 containing 0.3 % of TFA) and further purified by HPLC (column Atlantis® DC18 5 µm, 19x100

mm, flow: 12.5 mL/min $CH_3CN/H_2O$ 60:40 containing 1 % of TFA, v/v) to give **8** (215 mg, 81 %) as a white solid.

**mp**: 51-52 °C. **[α]$_D$**$^{18}$ = -12 (*c* 0.9, $CH_2Cl_2$). **$^1$H NMR** (400 MHz, $CDCl_3$) *δ* 9.77 (COOH); 7.79 (d, 2H, *J* = 7.5 Hz, 2 $H_{Fmoc}$); 7.64 (t, 2H, *J* = 6.4 Hz, 2 $H_{Fmoc}$); 7.43 (t, 2H, *J* = 7.5 Hz, 2 $H_{Fmoc}$); 7.34 (t, 2H, *J* = 7.4 Hz, 2 $H_{Fmoc}$); 7.20 (dd, 1H, *J* = 5.0, 1.3 Hz, H-$5_{Thiophene}$); 6.96-6.88 (m, 2H, H-$4_{Thiophene}$, H-$3_{Thiophene}$); 5.73 (d, 1H, *J* = 8.0 Hz, NH); 4.67 (ddd, *J* = 8.0, 6.0, 4,6 Hz, C*H*$CH_2$); 4.51-4.41 (m, 2H, 2 $CH_{2Fmoc}$); 4.26 (t, 1H, *J* = 7.1 Hz, $CH_{Fmoc}$); 3.96 (s, 2H, 2 $CH_2$T); 3.05 (dd, 1H, *J* = 14.1, 4.6 Hz, CHC*H*$_2$); 2.98 (dd, 1H, *J* = 14.1, 6.0 Hz, CHC*H*$_2$). **$^{13}$C APT NMR** (100 MHz, $CDCl_3$) *δ* 175.2 ($C_{COOH}$); 156.1 ($C_{Fmoc}$); 143.8 (C); 143.6 (C); 135.4 (C); 135.4 (C); 140.9 (C); 127.8 (2 $C_{Fmoc}$); 127.2 (2 $C_{Fmoc}$); 126.8 (2 $C_{Fmoc}$); 125.5 ($C_{Thiophene}$); 125.2 ($C_{Thiophene}$); 125.2 ($C_{Thiophene}$); 120.1 (2 $C_{Fmoc}$); 67.4 ($C_{Fmoc}$); 53.4 (*C*H$CH_2$); 47.1 ($C_{Fmoc}$); 33.3 (CH*C*H$_2$); 31.1 (*C*H$_2$-Thiophene). **Elemental analysis** calcd (%) for $C_{23}H_{21}NO_4S_2$: C, 62.85; H, 4.82; N, 3.19; O, 14.56; S, 14.59; found: C 62.12, H 4.96, N 3.20, S 14.99.

### 2.3. Synthesis of quinoline building block (12)

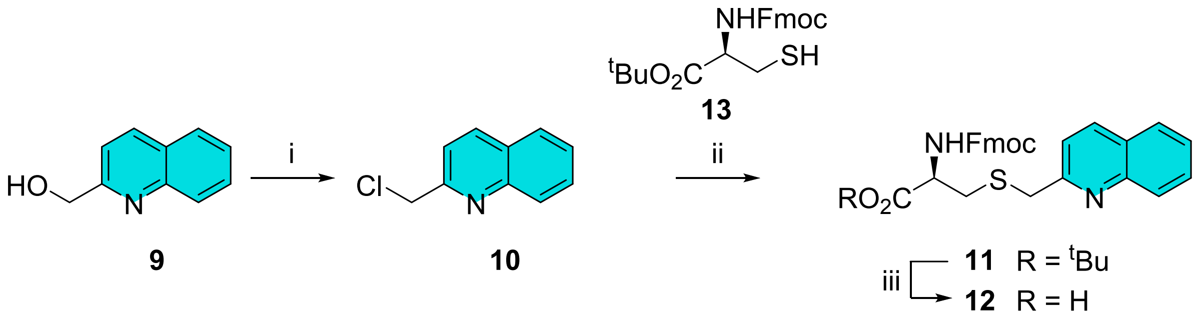


**Scheme S2**. Synthetic approach for the synthesis of building blocks **12**. Reagent and conditions: (i) $SOCl_2$, DCM, 0 °C to rt, 1 h, 85 %; (ii) **13**, $NaHCO_3$, $Bu_4NBr$, $H_2O$, EtOAc, 24 h, rt, 82 % (yield calculated on the limiting reagent **13**); (iii) TFA, DCM, 0 °C to rt, 2 h, 87 %.

### ***N*-Fmoc-*S*-methyl-2-quinolyl-L-cysteine *tert*-Butyl Ester (11)**

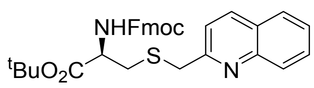


To a stirred solution of **9** (600 mg, 3.77 mmol) in anhydrous DCM (6 mL) cooled to 0 °C under Ar atmosphere, $SOCl_2$ (275 µL, 3.77 mmol) was added. The solution was stirred at room temperature for 1 hour, then diluted with DCM (50 mL) and washed with saturated $NaHCO_3$ aqueous solution (10 mL). and brine (10 mL). The organic phase was dried with anhydrous $MgSO_4$ and concentrated giving crude **10** (570 mg, 85 %) as a transparent liquid that was directly used in the next step without further purification.

To a stirred solution of **13** (485 mg, 1.21 mmol) in anhydrous DMF (1.5 mL) $K_2CO_3$ (420 mg, 3.03 mmol) was added, and the mixture was vigorously stirred at room temperature for 10 minutes. Then a solution of freshly prepared **10** (460 mg, 2.59 mmol) in anhydrous DMF (1.5

mL) was added and the resulting mixture was vigorously stirred at room temperature for 4 hours. The mixture was diluted with DCM (50 mL); washed with saturated $NaHCO_3$ aqueous solution (50 mL) and the aqueous layer back extracted with DCM (3 x 20 mL). The combined organic phases were dried with anhydrous $MgSO_4$ and concentrated. The crude residue was purified by column chromatography on silica gel (Hexane/EtOAc, 1:0 to 4:1 v/v) to give **11** (354 mg, 54 %, calculated on the limiting reagent **13**) as a white solid.

**mp:** 51-54 °C. **$[\alpha]_D$**$^{22}$ = -17 (*c* 1.0, $CH_2Cl_2$). **$^1$H NMR** (400 MHz, $CDCl_3$) $\delta$ 8.14 (t, 2H, $J$ = 8.5 Hz, 2 $H_{Quinol.}$); 7.79-7.72 (m, 3H, 2 $H_{Fmoc}$, $H_{Quinol.}$); 7.69 (ddd, 1H, $J$ = 8.5, 6.9, 1.5 Hz, $H_{Quinol.}$); 7.66-7.61 (m, 2H, $H_{Fmoc}$, $H_{Quinol.}$); 7.54-7.46 (m, 2H, $H_{Fmoc}$, $H_{Quinol.}$); 7.37 (t, 2H, $J$ = 7.4 Hz, 2 $H_{Fmoc}$); 7.30-7.23 (m, 2H, 2 $H_{Fmoc}$); 6.84 (d, 1H, $J$ = 7.7 Hz, NH); 4.58 (ddd, 1H, $J$ = 7.7, 6.6, 4.1 Hz, C*H*$CH_2$); 4.43-4.31 (m, 2H, 2 $CH_{2Fmoc}$); 4.24 (t, 1H, $J$ = 7.3 Hz, $CH_{Fmoc}$); 4.08 (d, 2H, $J$ = 3.1 Hz, 2 C$H_2$-Quinol.); 2.99 (dd, 1H, $J$ = 14.1, 4.1 Hz, CHC$H_2$);2.93 (dd, 1H, $J$ = 14.1, 6.6 Hz, CHC$H_2$); 1.44 (s, 9H, $^t$Bu). **$^{13}$C APT NMR** (100 MHz, $CDCl_3$) $\delta$ 169.8 ($C_{COO\mathit{t}\text{-}Bu}$); 158.3 (C); 156.0 (C); 144.0 (2 $C_{Fmoc}$); 143.9 (2 $C_{Fmoc}$); 141.3 (C); 137.5 (CH); 130.0 (CH); 128.8 (CH); 127.7 (2 CH); 127.6 (CH); 127.1 (CH); 127.1 (CH); 127.0 (C); 126.6 (CH); 125.3 (CH); 125.3 (CH); 121.4 (CH); 120.0 (2 CH); 82.5 (C); 67.1 ($C_{Fmoc}$); 55.3 (*C*H$CH_2$); 47.2 ($C_{Fmoc}$); 38.2 (*C*$H_2$-Quinol.); 33.3 (CH*C*$H_2$); 28.0 (3 $C_{\mathit{t}\text{-}Bu}$). **Elemental analysis** calcd (%) for $C_{32}H_{32}N_2O_4S$: C, 71.09; H, 5.97; N, 5.18; O, 11.84; S, 5.93; found: C 71.20, H 6.13, N 5.09, S 5.78.

***N*-Fmoc-*S*-methyl-2-quinolinyl-L-cysteine (12)**

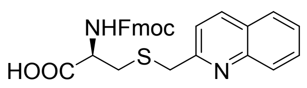


To a stirred solution of **11** (305 mg, 0.56 mmol) in anhydrous DCM (3.7 mL) cooled to 0 °C, TFA (3.7 mL 48.35 mmol) was added. The resulting solution was allowed to rise to room temperature, stirred for 2 hours, and then concentrated under reduced pressure. The residue was purified by column chromatography on silica gel (DCM/MeOH, 1:0 to 95:5 v/v) to give **12** (236 mg, 87 %) as a white solid. **mp**: 177-180 °C with decomposition. **$[\alpha]_D$**$^{21}$ = -8 (*c* 0.3, $CH_2Cl_2$). **$^1$H NMR** (400 MHz, $CDCl_3$) $\delta$ 11.11 (s, 1H, COOH); 8.69 (d, 1H, $J$ = 8.6 Hz, $H_{Quinol.}$); 8.48 (d, 1H, $J$ = 8.6 Hz, $H_{Quinol.}$); 8.04-7.96 (m, 3H, 3 $H_{Quinol.}$); 7.81 (t, 1H, $J$ = 7.6 Hz, $H_Q$); 7.74 (d, 2H, $J$ = 7.5 Hz, 2 $H_{Fmoc}$); 7.57 (t, 2H, $J$ = 6.7 Hz, 2 $H_{Fmoc}$); 7.38 (t, 2H, $J$ = 7.5 Hz, 2 $H_{Fmoc}$); 7.32-7.26 (m, 2H, 2 $H_{Fmoc}$); 6.08 (d, 1H, $J$ = 7.5 Hz, NH); 4.65-4.58 (m, 1H, C*H*$CH_2$); 4.53 (d, 1H, $J$ = 15.8 Hz, $CH_{2Fmoc}$); 4.33-4.19 (m, 3H, $CH_{2Fmoc}$, 2 C$H_2$-Quinol.); 4.13 (t, 1H, $J$ = 7.2 Hz, $CH_{Fmoc}$); 3.19 (dd, 1H, $J$ = 14.8, 4.5 Hz, CHC$H_2$); 2.90 (dd, 1H, $J$ = 14.8, 5.3 Hz, $CHCH_2$). **$^{13}$C APT NMR** (100 MHz, $CDCl_3$) $\delta$ 171.8 ($C_{COOH}$); 157.7 (C); 155.9 (C); 145.3 ($CH_Q$); 14.0 (C); 143.8 (C); 141.4 (2 C); 138.6 (C); 134.5 ($C_{Quinol.}$); 130.2 ($C_{Quinol.}$); 128.4 ($C_{Quinol.}$); 127.9 (2 $C_{Fmoc}$); 127.7 (C); 127.3 (2 $C_{Fmoc}$); 125.4 ($C_{Fmoc}$); 125.3 ($C_{Fmoc}$); 122.2 ($C_{Quinol.}$); 122.0 ($C_{Quinol.}$); 120.1 (2 $C_{Fmoc}$); 67.3 ($C_{Fmoc}$); 53.3 (*C*H$CH_2$); 47.2 ($C_{Fmoc}$); 35.2 (*C*$H_2$-Quinol.); 33.8 (CH*C*$H_2$). **Elemental analysis** calcd (%) for $C_{28}H_{24}N_2O_4S$: C, 69.40; H, 4.99; N, 5.78; O, 13.21; S, 6.62; found: C 69.63, H

4.93, N 5.69, S 6.54.

### 2.4 Solid phase peptide synthesis (2,3,4,18,19)

(Glyco)peptides were synthesized by stepwise microwave-assisted solid-phase peptide synthesis on a Liberty Blue synthesizer using the Fmoc strategy on Rink Amide MBHA resin (0.1 mmol). Fmoc-Thr[GalNAc(Ac)$_3$-α-D]-OH was synthesized as described in the literature.[9] This compound and the building blocks **8** and **12** (2.0 equiv) were manually coupled using HBTU [(2-(1*H*-benzotriazol-1-yl)-1,1,3,3-tetramethyluronium hexafluorophosphate] (0.9 equiv) and diisopropyl ethyl amine –DIPEA– (2.0 equiv) , while all other Fmoc amino acids (5.0 equiv) were automatically coupled using oxyma pure/DIC (*N*,*N*'-diisopropylcarbodiimide). The *O*-acetyl groups of GalNAc moiety were removed treating the resin-bound peptide with a mixture of $NH_2NH_2$/MeOH (7:3 v/v) 3x(5 mL, shaking for 30 minutes). (Glyco)peptides were then released from the resin, along with removal of the acid-sensitive sidechain protecting groups, using 3.55 mL of TFA/thioanisole/DODT/anisole (2.7:0.15:0.1:0.6, v:v:v:v) for 2 h at room temperature. (Glyco)peptides were then precipitated with cold diethyl ether (20 mL) and centrifuged for 5 min at 6500 rpm. The supernatant solution was discarded, and this process was repeated twice. Finally, (glyco)peptides were dried and redissolved in water to be purified by reverse phase HPLC on a Phenomenex Luna C18(2) column (10 µm, 250 mm x 21.2 mm). UV detection was done at 212 nm.

## Quinoline glycopeptide (2)

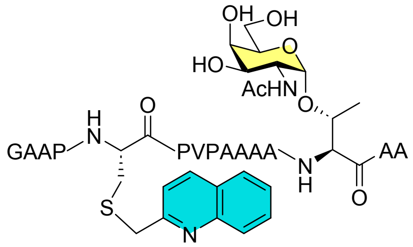


Glycopeptide **2** was synthesized according to the general method described in section 2.4 and purified on preparative HPLC using a linear gradient of $CH_3CN$/H2O ($H_2O$ phase containing 0.1% TFA) of 5/95 to 30/70 over 30 minutes with a flow rate of 20 mL/min.

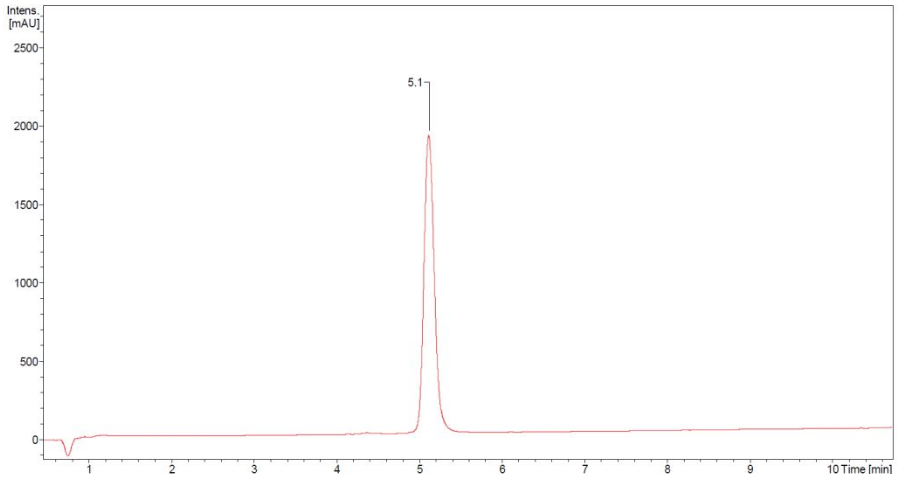


**Figure S2**. Analytical UPLC chromatogram of glycopeptide **2**, on a Phenomenex BioZen C18 column (1.7 μM, 100 mm x 2.1 mm), 30 °C, Rt = 5.1 min (linear gradient: acetonitrile 0.1% formic acid water/ H2O 0.1% formic acid, (5:95) → (40:60) over 10 min, λ = 214 nm, flow rate: 0.35 mL/min).

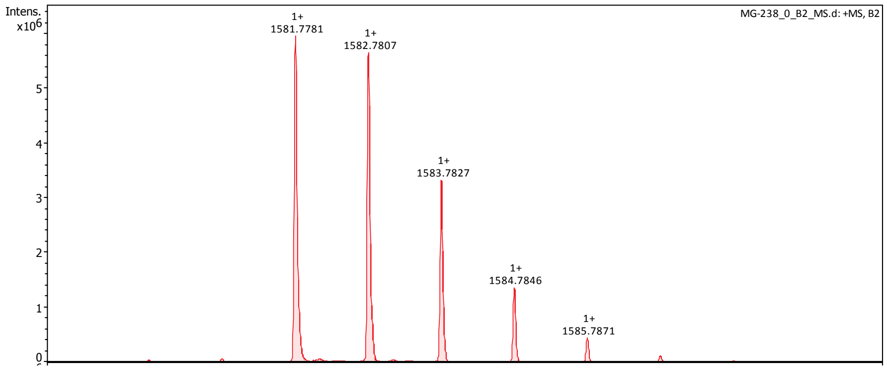


**Figure S3**. HRMS MALDI of glycopeptide **2**, formula: $C_{71}H_{109}N_{18}O_{21}S$ $[M+H]^+$ calculated: 1581.7730, found: 1581.7781.

**Thiophene glycopeptide (3)**

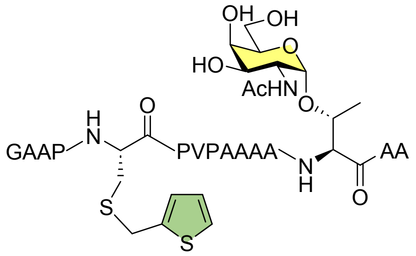


Glycopeptide **3** was synthesized according to the general method described in section 2.4 and purified on preparative HPLC using a linear gradient of $CH_3CN$/H2O ($H_2O$ phase containing 0.1% TFA) of 5/95 to 40/70 over 40 minutes with a flow rate of 20 mL/min.

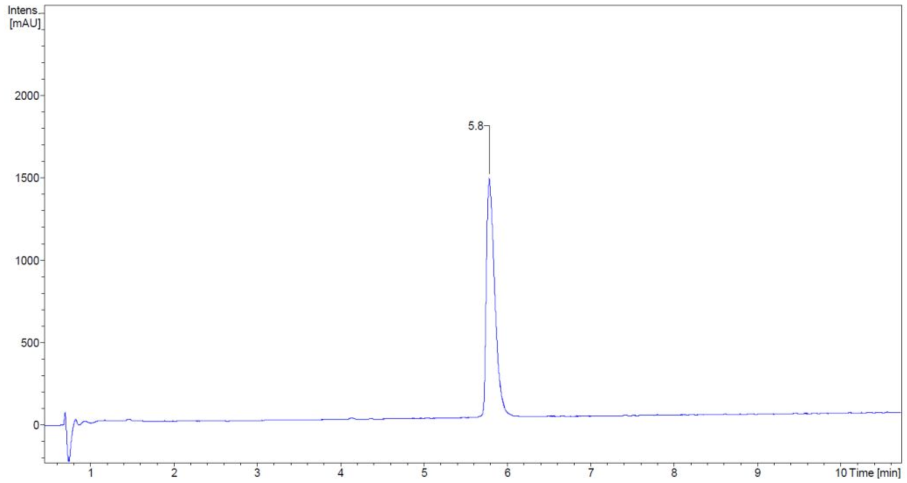


**Figure S4**. Analytical UPLC chromatogram of glycopeptide **3**, on a Phenomenex BioZen C18 column (1.7 µM, 100 mm x 2.1 mm), 30 °C, Rt = 5.8 min (linear gradient: acetonitrile 0.1% formic acid water/ H2O 0.1% formic acid, (5:95) → (40:60) over 10 min, λ = 214 nm, flow rate: 0.35 mL/min).

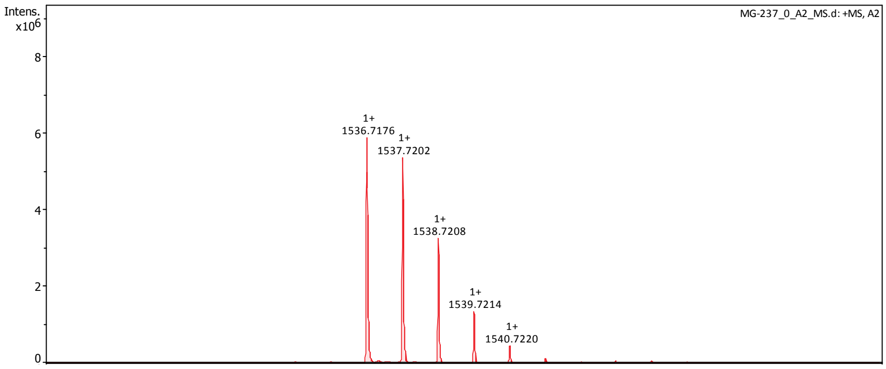


**Figure S5**. HRMS MALDI of glycopeptide **3**, formula: $C_{66}H_{106}N_{17}O_{21}S_2$ $[M+H]^+$ calculated: 1536.7185, found: 1536.7176.

## Negative control glycopeptide (4)

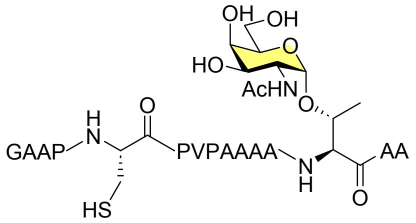


Glycopeptide **4** was synthesized according to the general method described in section 2.4 and purified on preparative HPLC using a linear gradient of $CH_3CN$/H2O ($H_2O$ phase containing 0.1% TFA) of 5/95 to 30/70 over 30 minutes with a flow rate of 20 mL/min.

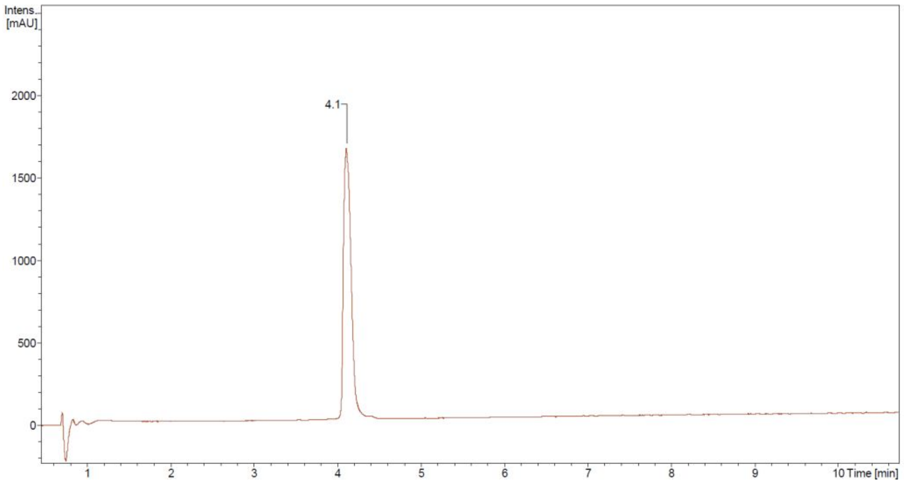


**Figure S6**. Analytical UPLC chromatogram of glycopeptide **4**, on a Phenomenex BioZen C18 column (1.7 µM, 100 mm x 2.1 mm), 30 °C, Rt = 4.1 min (linear gradient: acetonitrile 0.1% formic acid water/ H2O 0.1% formic acid, (5:95) → (40:60) over 10 min, λ = 214 nm, flow rate: 0.35 mL/min).

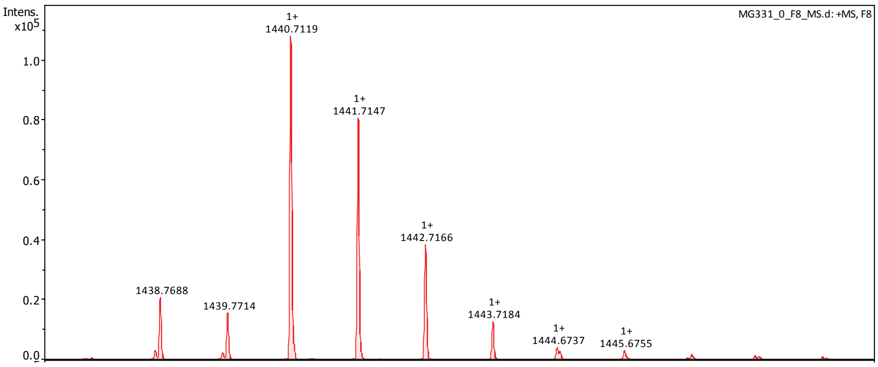


**Figure S7**. HRMS MALDI of glycopeptide **4**, formula: $C_{61}H_{102}N_{17}O_{21}S$ $[M+H]^+$ calculated: 1440.7151, found: 1440.7119.

## Thiophene glycopeptide (18)

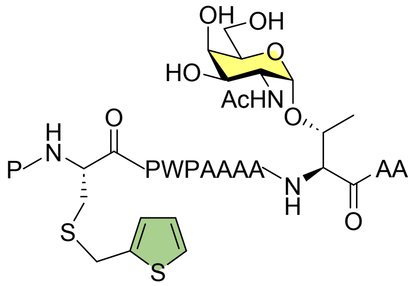


Glycopeptide **18** was synthesized according to the general method described in section 2.4 and purified on preparative HPLC using a linear gradient of $CH_3CN$/H2O ($H_2O$ phase containing 0.1% TFA) of 10/90 to 50/50 over 30 minutes with a flow rate of 20 mL/min.

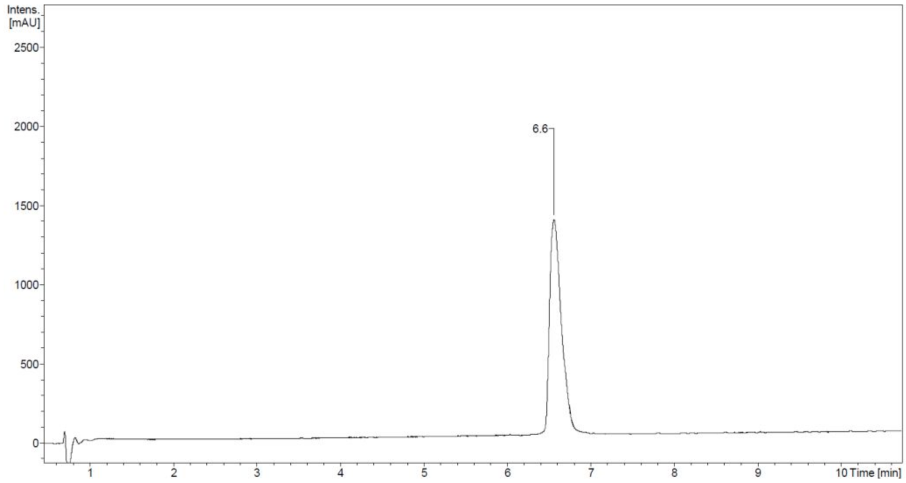


**Figure S8**. Analytical UPLC chromatogram of glycopeptide **18**, on a Phenomenex BioZen C18 column (1.7 μM, 100 mm x 2.1 mm), 30 °C, Rt = 6.6 min (linear gradient: acetonitrile 0.1% formic acid water/ H2O 0.1% formic acid, (5:95) → (40:60) over 10 min, λ = 214 nm, flow rate: 0.35 mL/min).

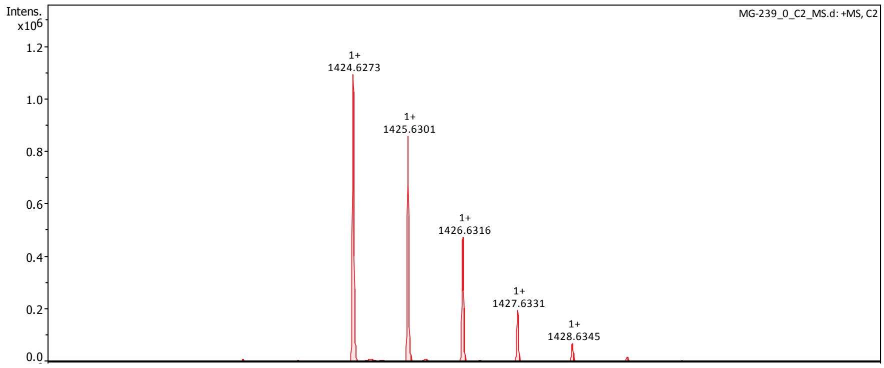


**Figure S9**. HRMS MALDI of glycopeptide **18**, formula: $C_{64}H_{94}N_{15}O_{18}S_2$ $[M+H]^+$ calculated: 1424.6337, found: 1424.6273.

## Thiophene glycopeptide (19)

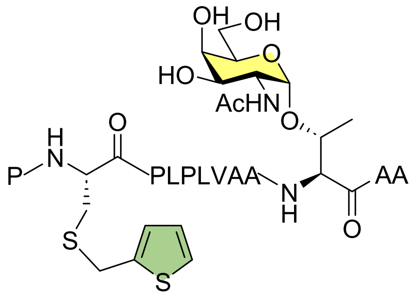


Glycopeptide **19** was synthesized according to the general method described in section 2.4 and purified on preparative HPLC using a linear gradient of $CH_3CN$/H2O ($H_2O$ phase containing 0.1% TFA) of 10/90 to 50/50 over 30 minutes with a flow rate of 20 mL/min.

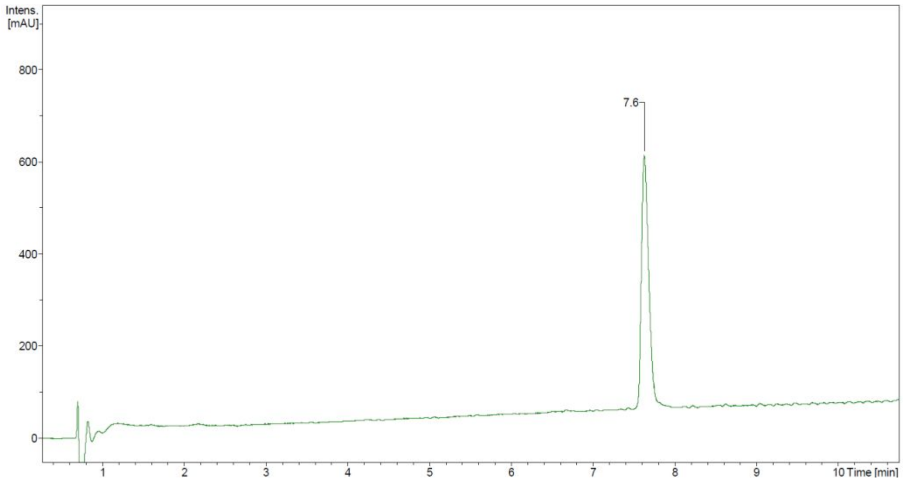


**Figure S10**. Analytical UPLC chromatogram of glycopeptide **19**, on a Phenomenex BioZen C18 column (1.7 µM, 100 mm x 2.1 mm), 30 °C, Rt = 7.6 min (linear gradient: acetonitrile 0.1% formic acid water/ H2O 0.1% formic acid, (5:95) → (40:60) over 10 min, λ = 214 nm, flow rate: 0.35 mL/min).

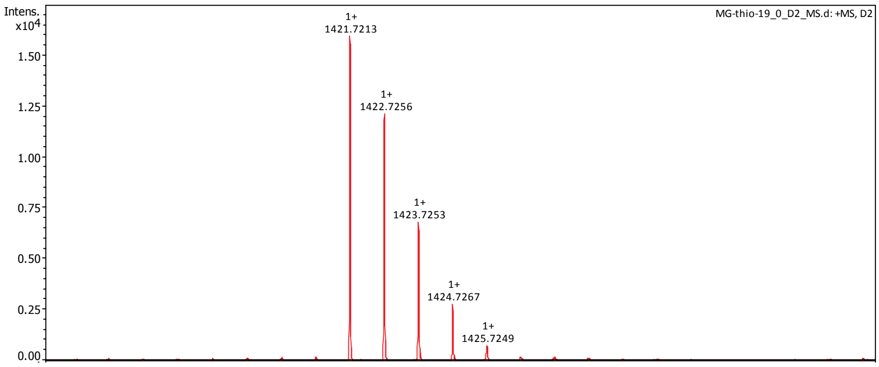


**Figure S11**. HRMS MALDI of glycopeptide **19**, formula: $C_{64}H_{105}N_{14}O_{18}S_2$ $[M+H]^+$ calculated: 1421.7167, found: 1421.7213.

## 2.5. NMR Spectra

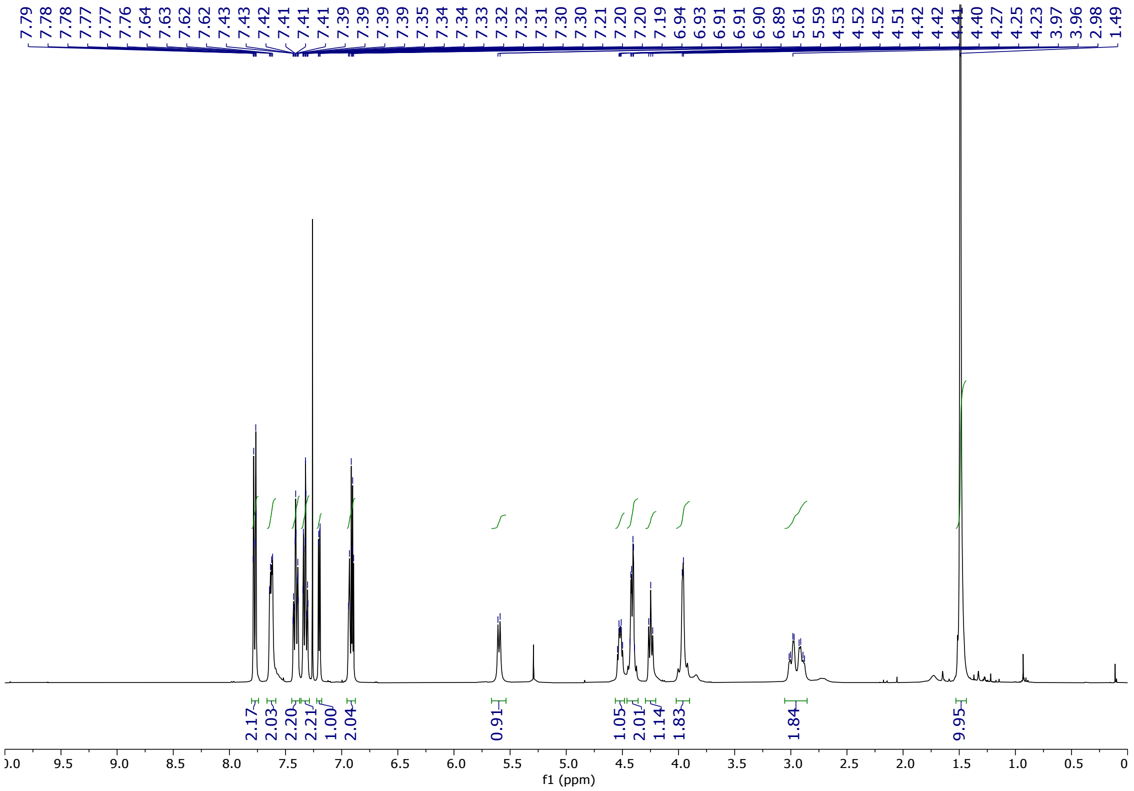


**Figure S12**. $^{1}H$ NMR (400 MHz, $CDCl_3$) spectrum of compound **7**.

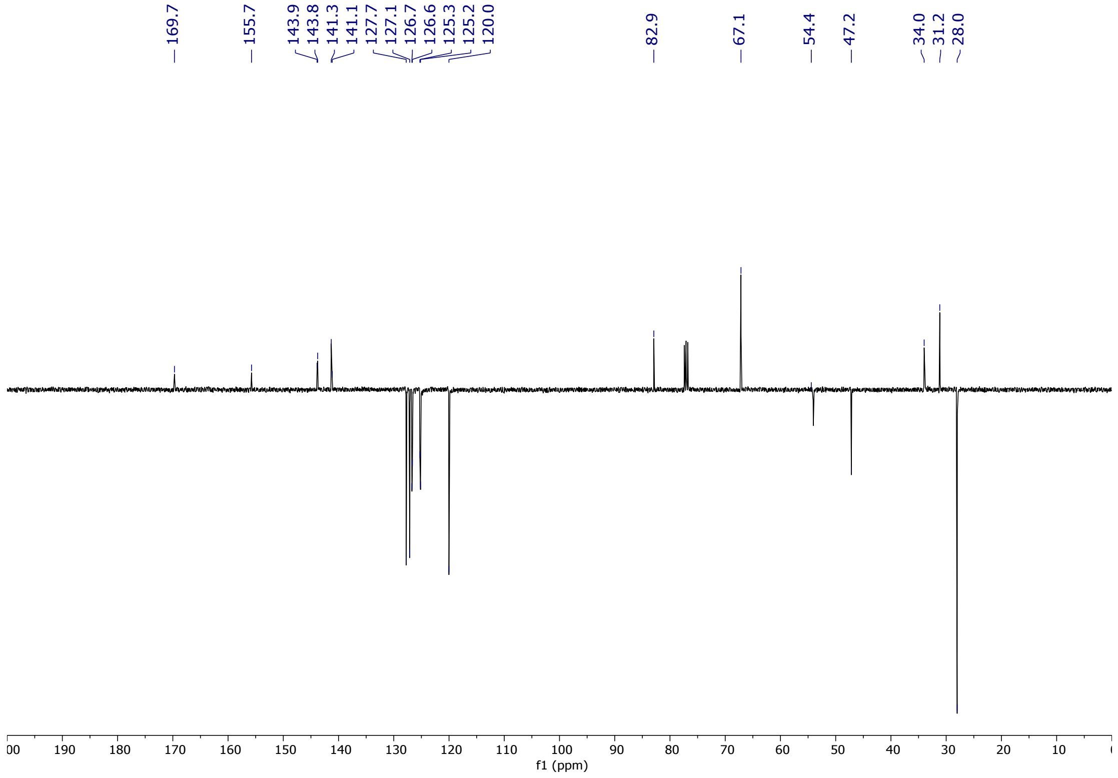


**Figure S13**. $^{13}C$ APT NMR (100 MHz, $CDCl_3$) spectrum of compound **7**.

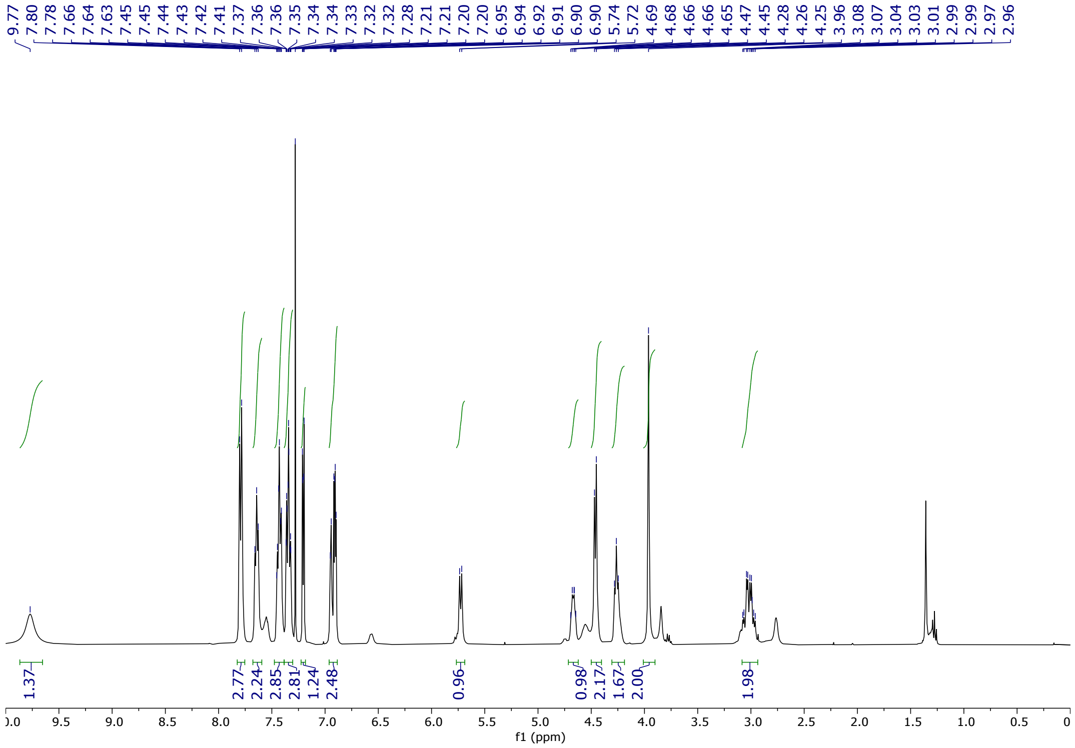


**Figure S14**. $^{1}H$ NMR (400 MHz, $CDCl_3$) spectrum of compound **8**.

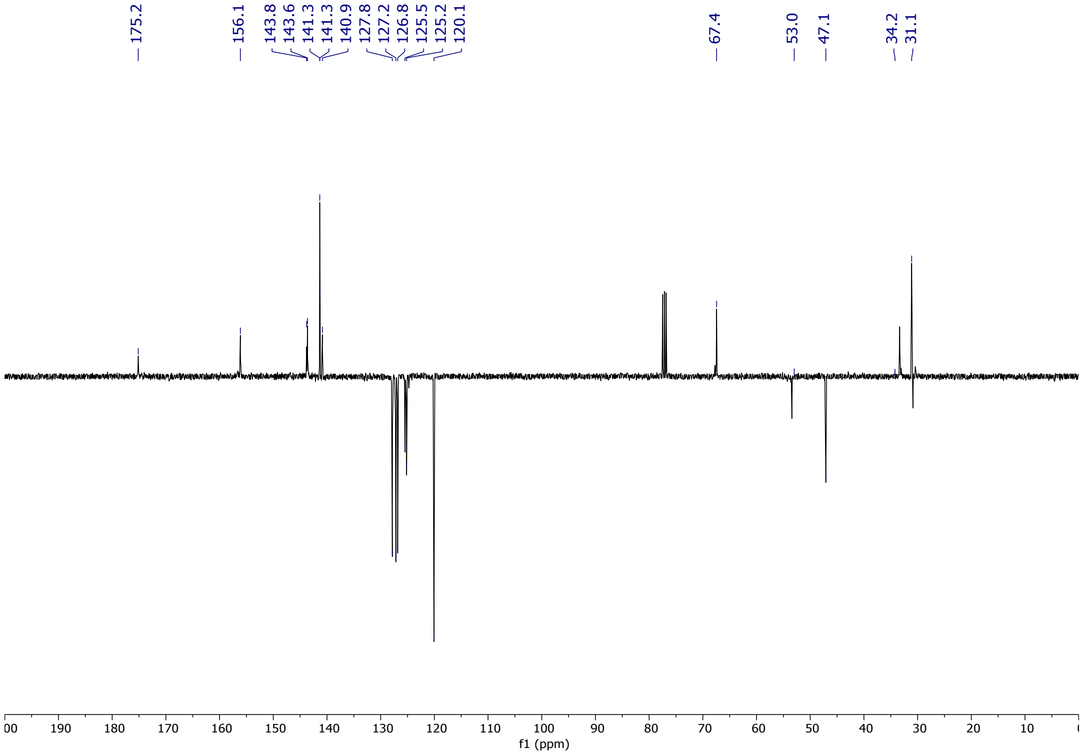


**Figure S15**. $^{13}C$ APT NMR (100 MHz, $CDCl_3$) spectrum of compound **8**.

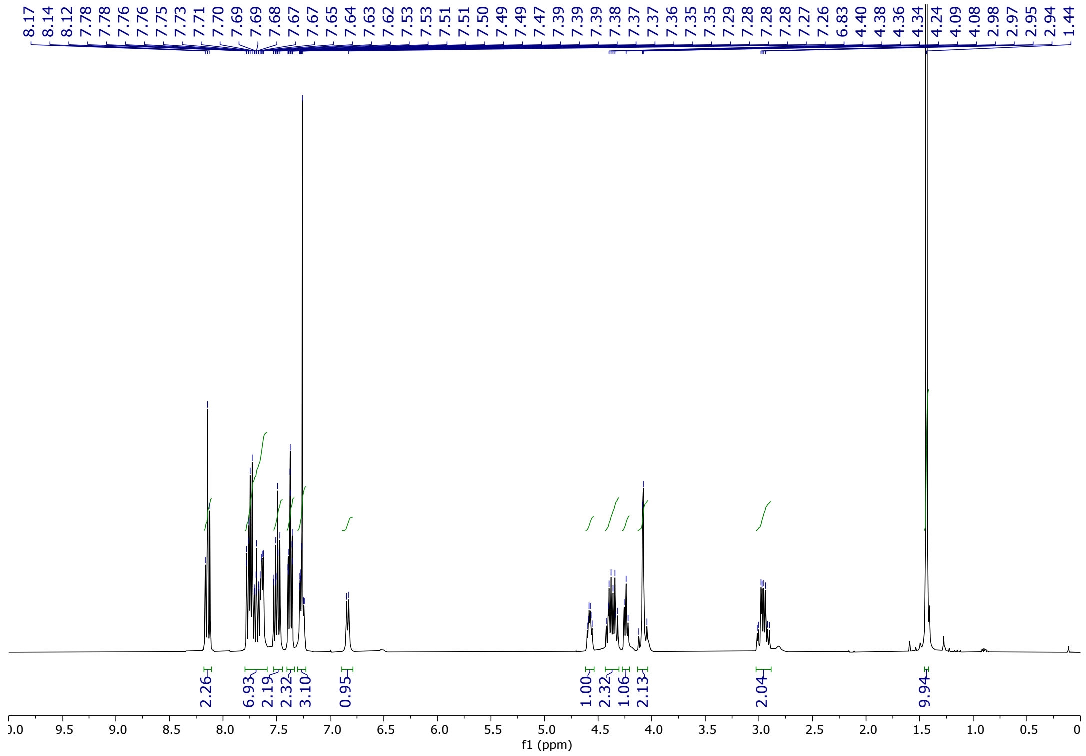


**Figure S16**. $^{1}H$ NMR (400 MHz, $CDCl_3$) spectrum of compound **11**.

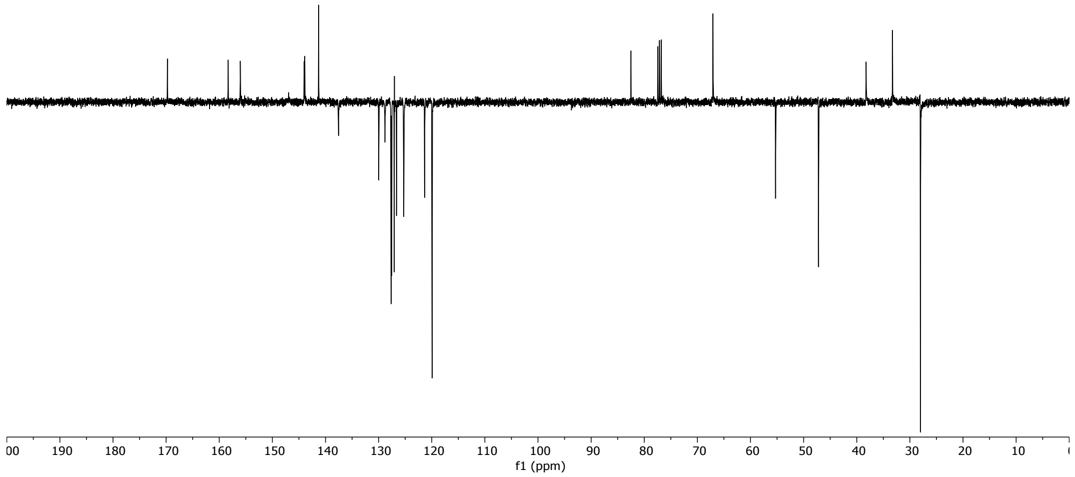


**Figure S17**. $^{13}C$ APT (100 MHz, $CDCl_3$) NMR spectrum of compound **11**.

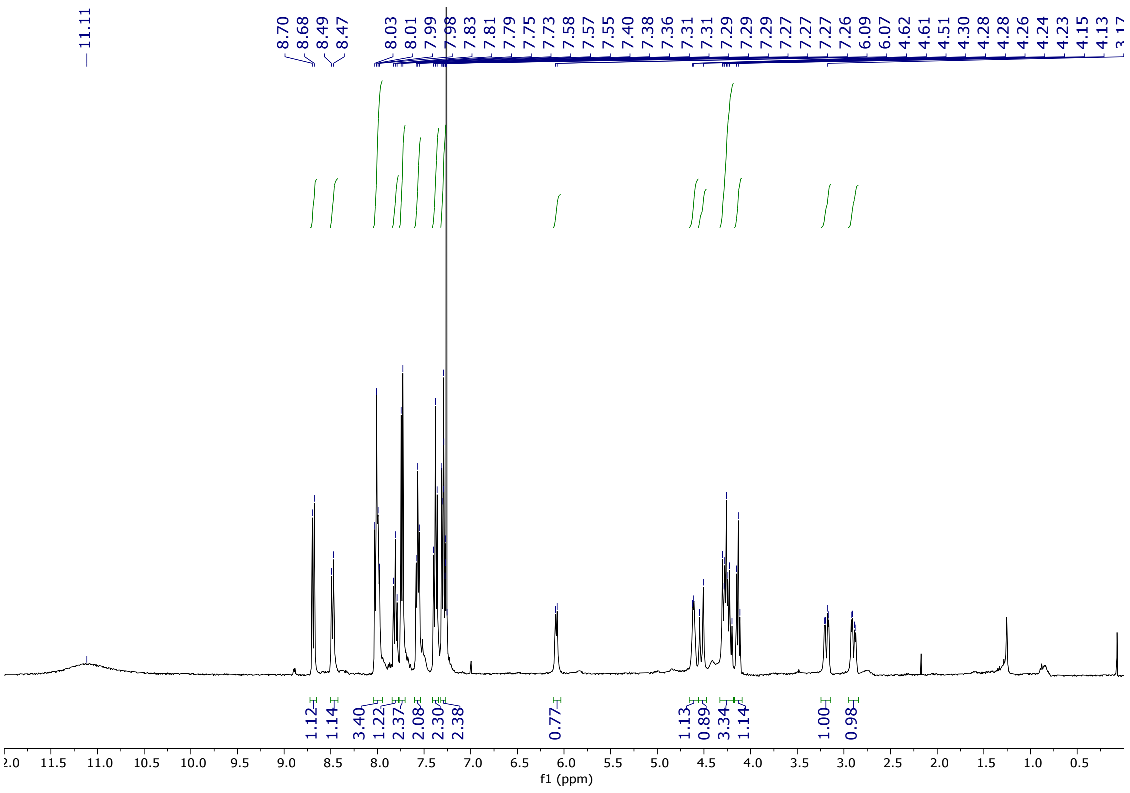


**Figure S18**. $^{1}H$ NMR (400 MHz, $CDCl_3$) spectrum of compound **12**.

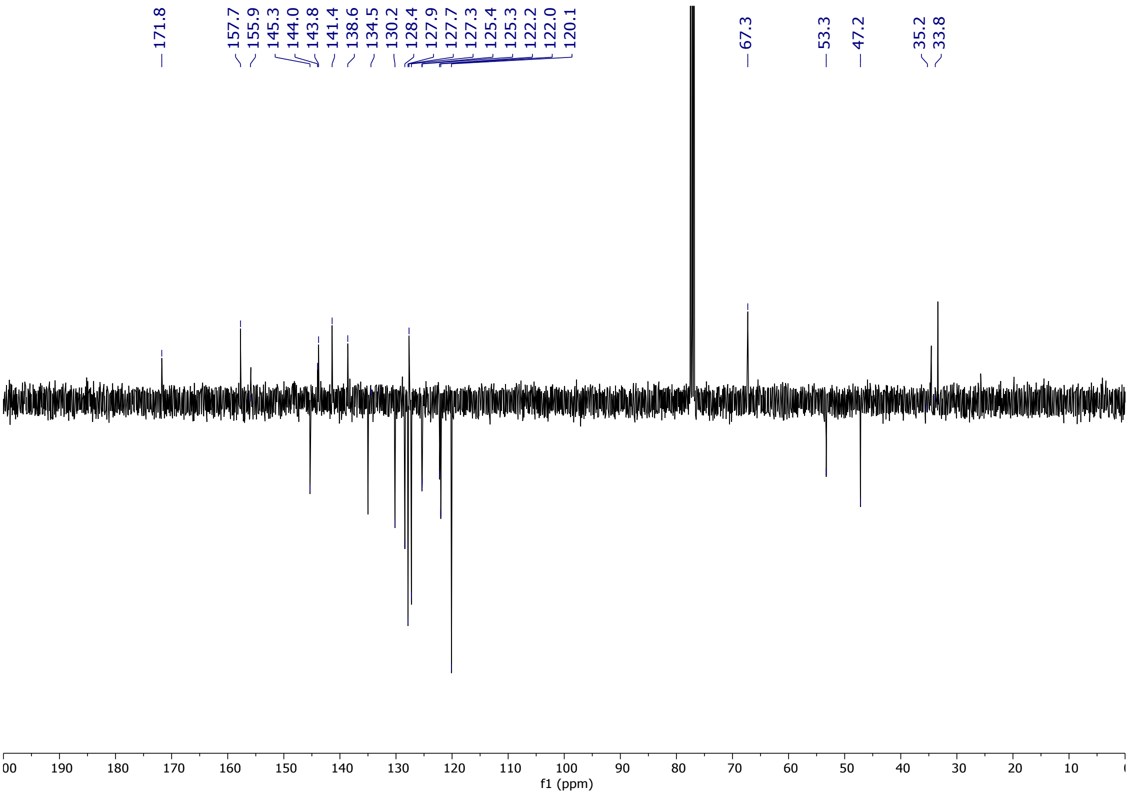


**Figure S19**. $^{13}C$ APT NMR (100 MHz, $CDCl_3$) spectrum of compound **12**.

**3. STD NMR STUDIES**

All experiments were performed at 278 K on a Bruker Avance III 800MHz spectrometer equipped with a 5-mm TXI 800MHz H-C/N-D-05 Z BTO probe. A compound **19** sample was prepared at 2 mM in 25 mM Tris-d11 pH 7.4, 100 mM NaCl, 1 mM DTT and 0.5 mM EDTA in $D_2O$ and assigned using standard COSY (cosydfesgpph), TOCSY (mlevphpr), $^{1}$H-$^{13}$C HSQC (hsqctgpsp) and NOESY (noesygpph) experiments. The residual water signal was used as a reference for chemical shifts.

For STD NMR experiments, the sample consisted of 1 mM compound **19** and 40 µM GalNAc-T2 (ligand: protein ratio 20:1) in the same deuterated buffer used for the assignment.

STD NMR experiments were performed using a train of 50 ms Gaussian pulses (0.4 mW, B1 field strength 78 Hz) applied on the f2 channel at -1 ppm (on-resonance) or 40 ppm (off resonance). A spoil sequence (2 trim pulses of 2.5 and 5ms followed by a 40 % z-gradient applied for 3ms at the beginning of the experiment) was used to destroy un anted x,y-magnetization from previous scan and a spinlock (1.55 W, 40 ms) was used to suppress protein signals (stddiff.3).

To obtain the binding epitopes of the ligands, STD NMR experiments were carried out at different saturation times, d20, (0.5, 1.0, 2.0, 3.0, 4.0, and 5.0 s) and the resulting building curves were fitted mathematically to a monoexponential equation, from which the initial slopes were obtained. From these values, the binding epitope was obtained by dividing all by the largest value, to which an arbitrary value of 100 % was assigned. STD NMR competition experiments were performed by adding equimolar concentration of UDP-GalNAc to the sample containing compound **19** and GalNAc-T2 and recording STD experiments for both samples at 2 s saturation time.

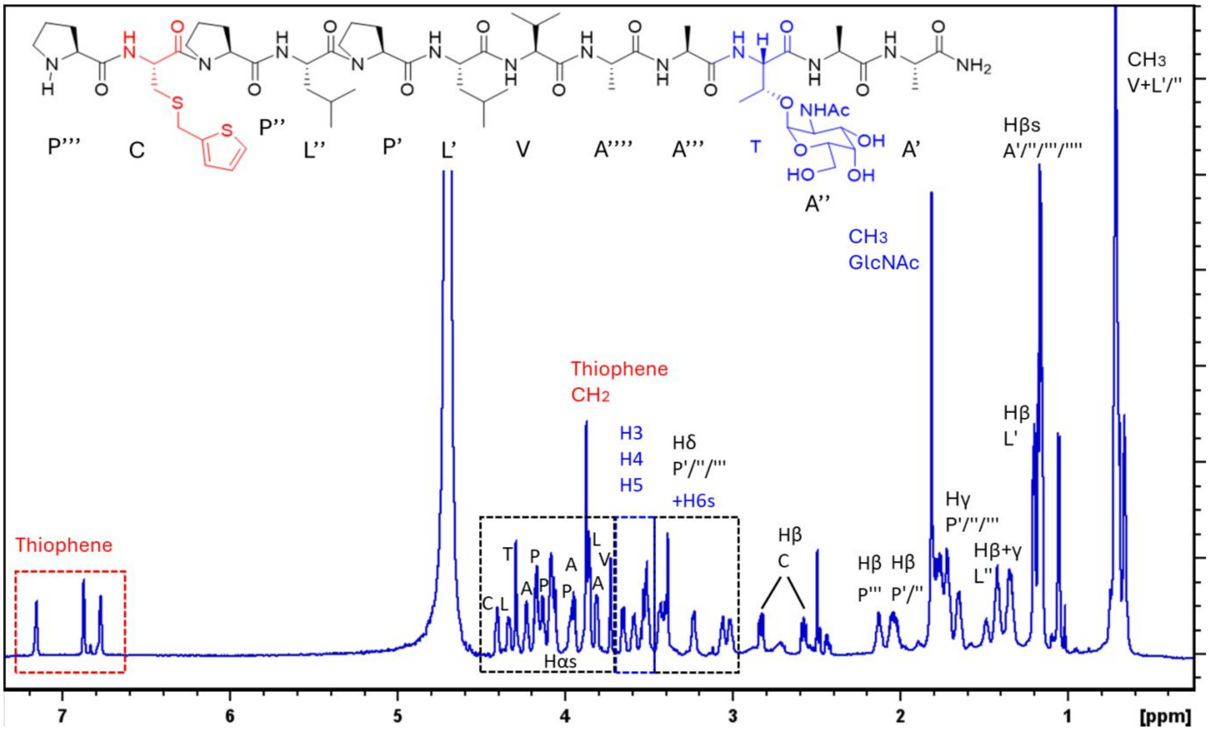


**Figure S20**. $^1H$ NMR (800MHz, $D_2O$) spectrum of compound **19** with proton assignment.

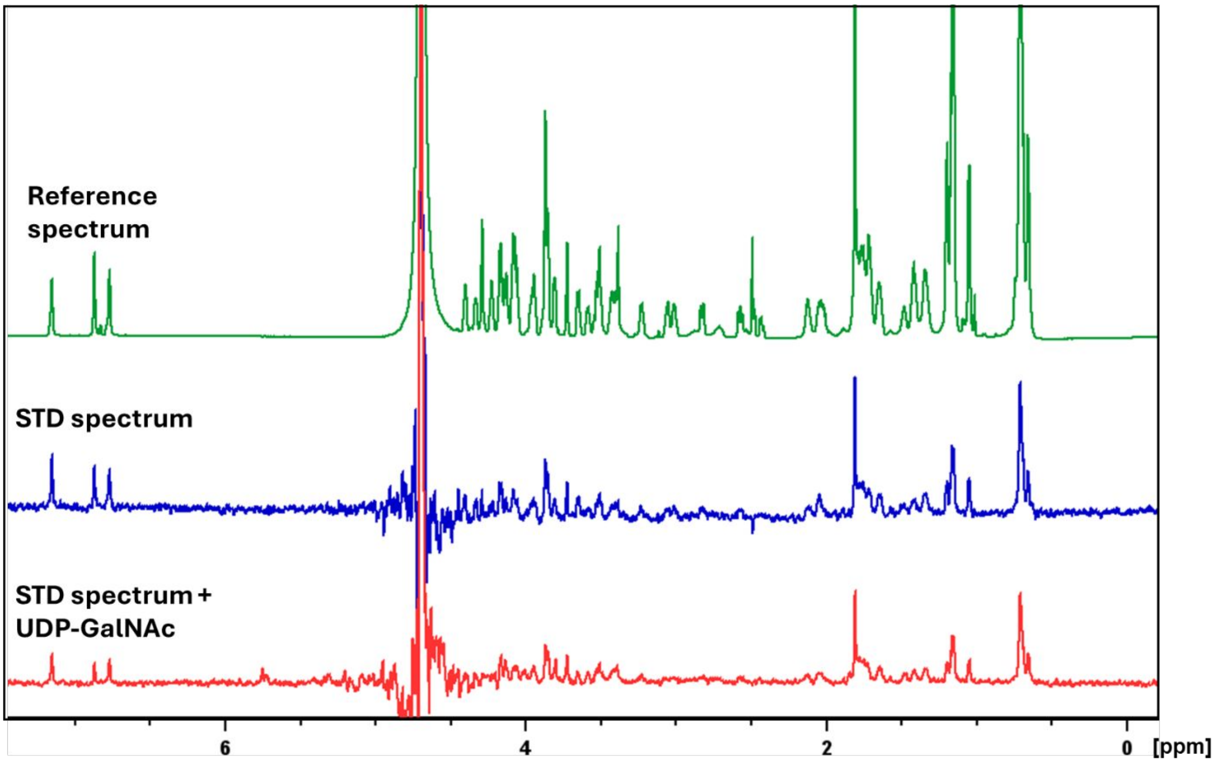


**Figure S21**. STD NMR (800MHz, in 25 mM Tris-d11 pH 7.4, 100 mM NaCl, 1 mM DTT and 0.5 mM EDTA in $D_2O$) competition experiment of compound **19** (1 mM) in complex with GalNAc-T2 (40 µM), and upon addition of UDP-GalNAc (1 mM).

## 4. BIOCHEMICAL STUDIES

### 4.1. Reagents and substrates

Stock solutions of inhibitor (5mM) and MUC1a substrate (4mM) were prepared from lyophilized powders. Additional serial dilutions of inhibitor were prepared of 1 mM, 200 µM, 50 µM, 10 µM, and 2 µM and the MUC1a substrate of 62.5 µM. Fully *N*-acetylated UDP-[$^3$H]GalNAc was obtained from American Radiolabeled Chemicals Inc (St. Louis, MO) whereas nonlabelled UDP-GalNAc was obtained from Millipore-Sigma (St. Louis, MO). A 0.2 mM stock solution of radiolabeled UDP-GalNAc was prepared by adding UDP-[$^3$H]GalNAc to unlabeled UDP-GalNAc to give ~6 x 108 DPM/µmole. ScintiVerse BD Cocktail fluid was obtained from Fisher Scientific (Pittsburgh, PA). Liquid scintillation counting was performed on a Beckman LS 6500 Scintillation Counter. BioPureSPN TARGA C18 macro spin columns were obtained from The Nest Group Inc. (Ipswich, MA).

### 4.2. GalNAc-Ts expression

Human GalNAc-T1 was a gift of Kelley Moremen (CCRC, University of Georgia) and expressed in HEK293F cells.[10] Human GalNAc-T2, and -T3 were obtained from Ramon Hertado-Guerrero (University of Zaragosa, SP) and expressed in *Pichia pastoris*.[11]

### 4.3. Inhibition reactions

The glycomimetic inhibitors were optimized using GalNAc-T2 activity assays as previously described.[12] The reactions were carried out in a total volume of 50 µl, containing 100 nM GalNAc-T2, 10 µM peptide MUC1a as substrate[13] and 20 µM UDP-[$^{14}$C]GalNAc (2000 cpm/nm) as the sugar donor. The designed glycomimetics were tested at a range of concentrations (0.2 µM to 500 µM): 500, 100, 20, 5, 1, 0.2, and 0 µM. The reactions were conducted in 25 mM cacodylate buffer (pH 7.4) with 10 mM $MnCl_2$ and 0.25% Triton X-100, and incubated at 22°C for 30 minutes. Excess UDP-[$^{14}$C]GalNAc was removed from the glycosylated peptide using DOWEX 1 chromatography, the product was subsequently analyzed through scintillation counting.

For the assays comparing the inhibition of GalNAc-T1, -T2 and -T3 by inhibitor **19** a similar protocol was used except UDP-[$^3$H]GalNAc was utilized and the products isolated on Targa C18 spin columns as previously described.[14] Final enzyme concentrations were 100 nM for GalNAc-T1 and -T3 and 200 nM for GalNAc-T2 while final MUC1a substrate and inhibitor **5** concentrations were as above. Reactions were incubated at 22°C for 30min for GalNAc-T1 and -T3 and 3h for GalNAc-T2. After incubation, reactions were quenched with 200µL of 0.5% TFA in $H_2O$ and passed through the spin columns. Columns were extensively washed with

0.1% TFA/$H_2O$ and glycopeptide products eluted with 0.1% TFA in 50/50 acetonitrile/$H_2O$ followed by 100% acetonitrile. The different eluates were analyzed by scintillation counting on a Beckman LS 6500 Scintillation Counter. Relative percent peptide glycosylation was calculated from the obtained $^3$H DPM as described in Ballard et al 2023. Reactions were repeated twice for each transferase. After subtracting the no substrate blank, under these conditions uninhibited glycosylation of MUC1a was 28% for GalNAc-T1, 4.5% for GalNAc-T2, and 3.2% for GalNAc-T3.

Percent activity was plotted against the log concentration of inhibitor **19** in μM. Graphs and $IC_{50}$ values were obtained using GraphPad Prism version 9.2 for windows and were fitted to the "log(inhibitor) vs. normalized response – Variable slope (four parameters)" model where the "baseline values" were taken as 100% inhibition at an inhibitor concentration at log(X) = 4 (i.e. $10^4$ μM).

### 4.4. Kinetic analysis

The $K_I$ values for the GalNAc-T1 T2 and T3 enzymes with respect to the MUC1a and UDP-GalNAc substrates was calculated rearranging the equation reported by Cheng & Prusoff (equation 19)[15] where $K_I = IC_{50} / ((1+ [A] / K_m^{UDP\text{-}GalNAc}) (1 + [B] / K_m^{MUC1a}))$ where a represents UDP-GalNAc and B represents the MUC1a substrate. The $K_m$ values were retrieved form the study reported by Wandall H. H. *et al.*,[16] which results are summarized in the table S1.

**Table S1.** Kinetic parameters calculated for GalNAc-T1, T2, and T3 using the MUC1a and UDP-GalNAc substrates.

| | $K_m^{(MUC1a)}$ | $K_m^{(UDP\text{-}GalNAc)}$ | $K_I$ |
|---|---|---|---|
| **GalNAc-T1** | 0.66 mM | 62 μM | 668 μM |
| **GalNAc-T2** | 2.13 mM | 10 μM | 21.3 μM |
| **GalNAc-T3** | 0.09 mM | 29 μM | 154 μM |

## 5. SUPPLEMENTARY REFERENCES


[1] D.A. Case, H.M. Aktulga, K. Belfon, I.Y. Ben-Shalom, J.T. Berryman, S.R. Brozell, D.S. Cerutti, T.E. Cheatham, III, G.A. Cisneros, V.W.D. Cruzeiro, T.A. Darden, R.E. Duke, G. Giambasu, M.K. Gilson, H. Gohlke, A.W. Goetz, R. Harris, S. Izadi, S.A. Izmailov, K. Kasavajhala, M.C. Kaymak, E. King, A. Ko- valenko, T. Kurtzman, T.S. Lee, S. LeGrand, P. Li, C. Lin, J. Liu, T. Luchko, R. Luo, M. Machado, V. Man, M. Manathunga, K.M. Merz, Y. Miao, O. Mikhailovskii, G. Monard, H. Nguyen, K.A. O'Hearn, A. Onufriev, F. Pan, S. Pantano, R. Qi, A. Rahnamoun, D.R. Roe, A. Roitberg, C. Sagui, S. Schott-Verdugo, A. Shajan, J. Shen, C.L. Simmerling, N.R. Skrynnikov, J. Smith, J. Swails, R.C. Walker, J. Wang, J. Wang, H. Wei, R.M. Wolf, X. Wu, Y. Xiong, Y. Xue, D.M. York, S. Zhao, P.A. Kollman, Amber 2022, University of California, San Francisco, **2022**.

[2] J. A. Maier, C. Martinez, K. Kasavajhala, L. Wickstrom, K. E. Hauser, C. Simmerling, *J. Chem. Theory Comput.* **2015**, *11*, 3696–3713.

[3] J. Wang, R. M. Wolf, J. W. Caldwell, P. A. Kollman, D. A. Case, *Journal of Computational Chemistry* **2004**, *25*, 1157–1174.

[4] K. N. Kirschner, A. B. Yongye, S. M. Tschampel, J. González-Outeiriño, C. R. Daniels, B. L. Foley, R. J. Woods, *Journal of Computational Chemistry* **2008**, *29*, 622–655.

[5] A. Jakalian, D. B. Jack, C. I. Bayly, *Journal of Computational Chemistry* **2002**, *23*, 1623–1641.

[6] E. Lira-Navarrete, M. de las Rivas, I. Compañón, M. C. Pallarés, Y. Kong, J. Iglesias-Fernández, G. J. L. Bernardes, J. M. Peregrina, C. Rovira, P. Bernadó, P. Bruscolini, H. Clausen, A. Lostao, F. Corzana, R. Hurtado-Guerrero, *Nat Commun* **2015**, *6*, 6937.

[7] W. L. Jorgensen, J. Chandrasekhar, J. D. Madura, R. W. Impey, M. L. Klein, *The Journal of Chemical Physics* **1983**, *79*, 926–935.

[8] T. Darden, D. York, L. Pedersen, *The Journal of Chemical Physics* **1993**, *98*, 10089–10092.

[9] C. Plattner, M. Höfener, N. Sewald, *Org. Lett.* **2011**, *13*, 545–547.

[10] K. W. Moremen, A. Ramiah, M. Stuart, J. Steel, L. Meng, F. Forouhar, H. A. Moniz, G. Gahlay, Z. Gao, D. Chapla, S. Wang, J.-Y. Yang, P. K. Prabhakar, R. Johnson, M. dela Rosa, C. Geisler, A. V. Nairn, J. Seetharaman, S.-C. Wu, L. Tong, H. J. Gilbert, J. LaBaer, D. L. Jarvis, *Nat Chem Biol* **2018**, *14*, 156–162.

[11] M. de las Rivas, E. J. Paul Daniel, Y. Narimatsu, I. Compañón, K. Kato, P. Hermosilla, A. Thureau, L. Ceballos-Laita, H. Coelho, P. Bernadó, F. Marcelo, L. Hansen, R. Maeda, A. Lostao, F. Corzana, H. Clausen, T. A. Gerken, R. Hurtado-Guerrero, *Nat Chem Biol* **2020**, *16*, 351–360.

[12] M. de las Rivas, H. Coelho, A. Diniz, E. Lira-Navarrete, I. Compañón, J. Jiménez-Barbero, K. T. Schjoldager, E. P. Bennett, S. Y. Vakhrushev, H. Clausen, F. Corzana, F. Marcelo, R. Hurtado-Guerrero, *Chemistry – A European Journal* **2018**, *24*, 8382–8392.

[13] H. H. Wandall, F. Irazoqui, M. A. Tarp, E. P. Bennett, U. Mandel, H. Takeuchi, K. Kato, T. Irimura, G. Suryanarayanan, M. A. Hollingsworth, H. Clausen, *Glycobiology* **2007**, *17*, 374–387.

[14] C. J. Ballard, M. R. Paserba, E. J. P. Daniel, R. Hurtado-Guerrero, T. A. Gerken, *Glycobiology* **2023**, cwad066.

[15] C. Yung-Chi, W. H. Prusoff, *Biochemical Pharmacology* **1973**, *22*, 3099–3108.

[16] H. H. Wandall, H. Hassan, E. Mirgorodskaya, A. K. Kristensen, P. Roepstorff, E. P. Bennett, P. A. Nielsen, M. A. Hollingsworth, J. Burchell, J. Taylor-Papadimitriou, H. Clausen, *Journal of Biological Chemistry* **1997**, *272*, 23503–23514.
